# DNA‐Intercalative Platinum Anticancer Complexes Photoactivated by Visible Light

**DOI:** 10.1002/chem.202101168

**Published:** 2021-05-27

**Authors:** Huayun Shi, Jana Kasparkova, Clément Soulié, Guy J. Clarkson, Cinzia Imberti, Olga Novakova, Martin J. Paterson, Viktor Brabec, Peter J. Sadler

**Affiliations:** ^1^ Department of Chemistry University of Warwick Coventry CV4 7AL UK; ^2^ Institute of Biophysics Czech Academy of Sciences Kralovopolska 135 61265 Brno Czech Republic; ^3^ Institute of Chemical Sciences School of Engineering & Physical Sciences Heriot-Watt University Edinburgh EH14 4AS UK

**Keywords:** anticancer activity, DFT calculations, DNA intercalation, interstrand crosslinking, photoactive platinum complexes

## Abstract

Photoactivatable agents offer the prospect of highly selective cancer therapy with low side effects and novel mechanisms of action that can combat current drug resistance. 1,8‐Naphthalimides with their extended π system can behave as light‐harvesting groups, fluorescent probes and DNA intercalators. We conjugated *N*‐(carboxymethyl)‐1,8‐naphthalimide (gly‐R‐Nap) with an R substituent on the naphthyl group to photoactive diazido Pt^IV^ complexes to form *t,t,t*‐[Pt(py)_2_(N_3_)_2_(OH)(gly‐R‐Nap)], R=H (**1**), 3‐NO_2_ (**2**) or 4‐NMe_2_ (**3**). They show enhanced photo‐oxidation, cellular accumulation and promising photo‐cytotoxicity in human A2780 ovarian, A549 lung and PC3 prostate cancer cells with visible light activation, and low dark cytotoxicity. Complexes **1** and **2** exhibit pre‐intercalation into DNA, resulting in enhanced photo‐induced DNA crosslinking. Complex **3** has a red‐shifted absorption band at 450 nm, allowing photoactivation and photo‐cytotoxicity with green light.

## Graphical abstract

**Green to go**: Photoactive all‐*trans* diazido Pt^IV^ complexes with an axial 1,8‐naphthalimide ligand that can pre‐intercalate into DNA have been synthesised. They can be photoactivated with visible light to generate Pt^II^ DNA interstrand crosslinks and cytotoxic ROS, and are thus promising photo‐chemotherapeutic agents. Notably, a 4‐NMe_2_ substituent red‐shifted absorption of the naphthalimide ring, giving rise to its green‐light photo‐cytotoxicity.

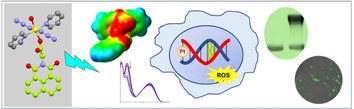

## Introduction

Phototherapy has attracted increasing attention in the clinic, owing to its cancer selectivity and novel mechanisms of action.^[1]^ However, the oxygen‐dependence of clinically approved photodynamic therapy (PDT) limits its applications in hypoxic tumours.^[1b]^ Photoactivated chemotherapy (PACT) agents, in contrast, exert photo‐cytotoxicity independently of oxygen.^[1b]^ Diazido Pt^IV^ complexes are promising PACT agents that are dark‐stable, but decompose upon irradiation to generate DNA binding Pt^II^ species, azidyl radicals and ROS, showing high photo‐cytotoxicity.^[2]^ The first diazido Pt^IV^ complexes *cis*,*trans*‐[Pt(en)(N_3_)_2_(OH)_2_] and *cis*,*cis*,*trans*‐[Pt(NH_3_)_2_(N_3_)_2_(OH)_2_] reported in 2003 exhibited photo‐cytotoxicity upon irradiation with UVA light.^[3a]^ Interestingly, Pt^IV^ complexes with two azide ligands in *trans* positions have higher aqueous solubility, and a more intense and red‐shifted ligand‐to‐metal charge‐transfer (LMCT) band compared with their *cis* isomers.^[3b]^ Notably, higher photo‐cytotoxicity was also detected for the all *trans* complexes with UVA irradiation. The replacement of NH_3_ by pyridine can also red‐shift the LMCT absorption and improve the photo‐cytotoxicity.^[3c]^ Complex *trans,trans,trans*‐[Pt(py)_2_(N_3_)_2_(OH)_2_] (**FM‐190**) shows photo‐cytotoxicity with blue light and is a promising candidate for further modification to achieve higher photo‐cytotoxicity, cancer selectivity, and red‐shifted activation.[^2^, ^3d^] The conjugation of diazido Pt^IV^ complexes to up‐conversion nanoparticles (UCNPs) allows photoactivation with near infrared light, but the longest excitation wavelength for reported simple diazido Pt^IV^ complexes to exert photo‐cytotoxicity is 465 nm until now.[^2^, ^4^] Longer wavelength excitation allows deeper tissue penetration, and therefore potentially more efficient photo‐cytotoxicity in tissues.^[5]^

1,8‐Naphthalimides with their extended π‐conjugated system are well‐known versatile fluorescent molecules that have been widely used in anticancer treatment.^[6]^ Substitution and aromatic ring extension can influence the photochemical and photophysical properties of 1,8‐naphthalimides effectively.[^6^, ^7^] The highly potent naphthalimides Mitonafide and Amonafide, have entered clinical trials for treatment of various tumours.^[8]^ The conjugation of 1,8‐naphthalimides with anticancer complexes enhances their cytotoxicity and enables cellular imaging.[^7^, ^9^] Pt complexes containing 1,8‐naphthalimides exhibit dual DNA binding modes and enhanced cytotoxicity.^[10]^ 1,8‐Naphthalimides can also be photosensitisers which generate ROS,^[6c]^ thus improving photo‐cytotoxicity when appended to photoactive complexes.^[11]^

**Figure 1:**
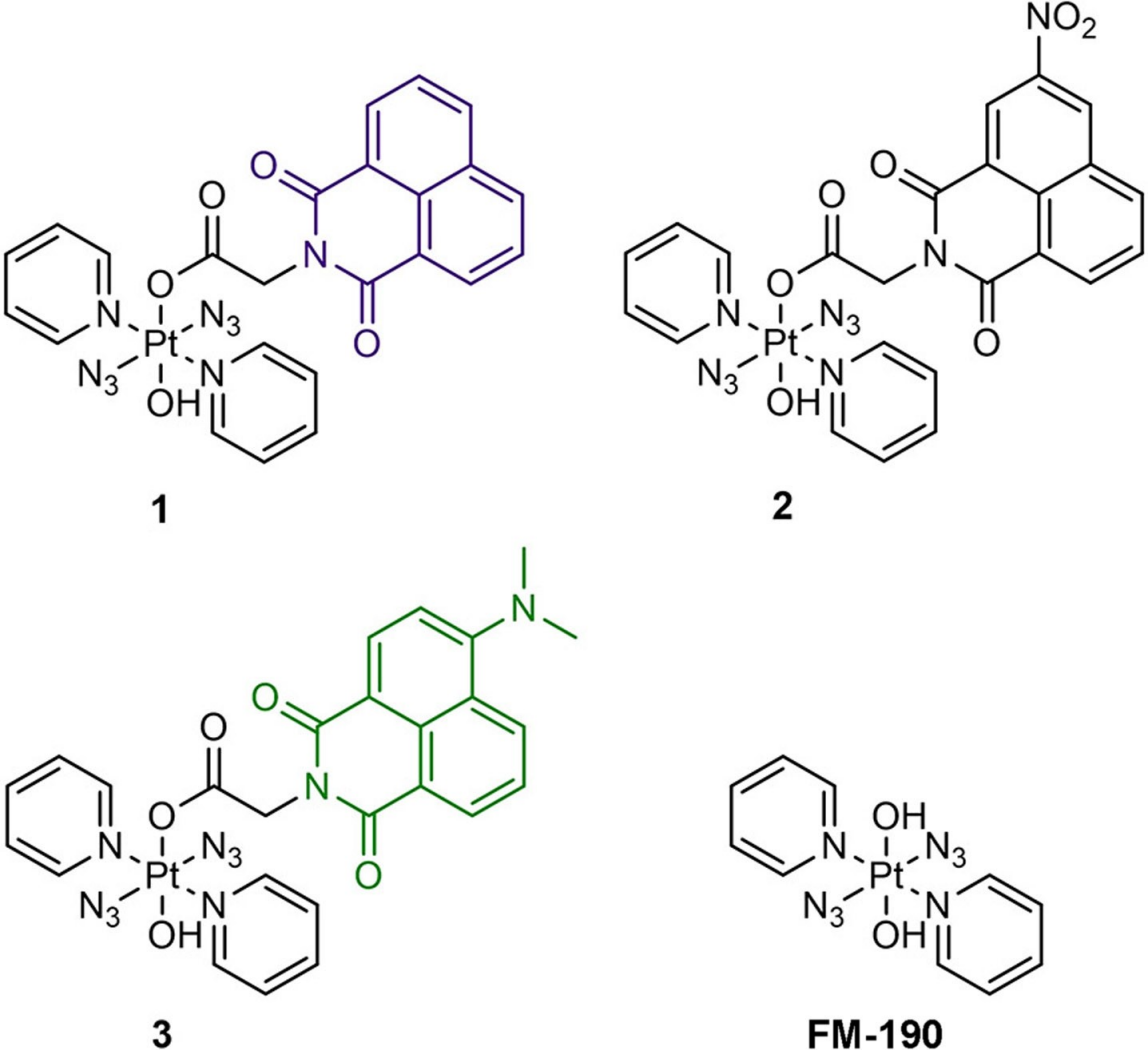
*Trans,trans,trans*‐[Pt(py)_2_(N_3_)_2_(OH)(gly‐R‐Nap)], R=H (**1**), 3‐NO_2_ (**2**) or 4‐NMe_2_ (**3**), complexes containing conjugated 1,8‐naphthalimide ligands studied in this work and the parent complex **FM‐190**.

In this work, we have synthesised and characterised three photoactive diazido Pt^IV^ complexes with axial fluorescent 1,8‐naphthalimide ligands, *trans,trans,trans*‐[Pt(py)_2_(N_3_)_2_(OH)(gly‐R‐Nap)], R=H (complex **1**), 3‐NO_2_ (**2**) or 4‐NMe_2_ (**3**; Figure 1). Since the optical properties of 1,8‐naphthalimides are sensitive to substitution in the aromatic ring, unsubstituted 1,8‐naphthalimide (**1**) and those with electron‐withdrawing −NO_2_ (**2**) or electron‐donating ‐NMe_2_ (**3**) substituents are compared. The crystal structures of the three complexes have been determined by X‐ray diffraction. Theoretical absorption spectra are compared with the observed spectra to assign the bands. The dark stability, photo‐decomposition, photo‐reactions with NADH and 5’‐GMP, DNA intercalation and crosslinking, photo‐cytotoxicity and cellular accumulation, and ROS generation upon irradiation have been investigated. The introduction of the 1,8‐naphthalimides can allow DNA‐targeting, red‐shifted excitation wavelengths, and improve the photo‐cytotoxicity of diazido Pt^IV^ complexes.

## Results and Discussion

### Synthesis and characterisation of complexes 1–3

Complexes **1**–**3** containing conjugated 1,8‐naphthalimide ligands were synthesised by coupling *trans,trans,trans*‐[Pt(py)_2_(N_3_)_2_(OH)(CO_2_CH_2_NH_2_)] (**Pt‐gly‐NH_2_**), containing an axial glycine, with the corresponding anhydride at 353 K. They have high HPLC purity (>95 %, Figure S1 in the Supporting Information), and were characterised by ESI‐HRMS and NMR spectroscopy (Figures S2–S7). In ^1^H NMR spectra, Pt‐coordinated pyridine has characteristic doublets with ^195^Pt satellites at around 8.8 ppm, and triplets at around 8.1 and 7.7 ppm, and ^13^C NMR resonances at *ca*. 149, 142 and 126 ppm. 1,8‐Naphthalimide ligands give ^1^H NMR peaks in the range 8.6 to 7.1 ppm and ^13^C NMR peaks from 135 to 113 ppm. 1,8‐Naphthalimide ligands **L1**‐**L3** were prepared for comparison using modified literature methods (Figures S8–S14).^[12]^ All complexes display a high‐energy band at around 260 nm, and an absorption maximum at around 300 nm (Figure S15a, Table S1). A shoulder at around 340 nm is observed for **1** and **2**, and an intense band at 450 nm for **3**. In contrast, no absorbance at 300 nm is observed for ligands **L1**–**L3** (Figure S15c, Table S1). However, peaks at around 340 nm for **1** and **2**, and 450 nm for **3**, were detected. Upon excitation at *λ*=355 nm, complex **1** exhibited purple emission (*λ*_max_=395 nm) with a quantum yield of 0.002; complex **3** exhibited green emission (*λ*_max_=546 nm) upon excitation at *λ*=450 nm, and a quantum yield of 0.006 (Figure S15b, Table S1). Similar emission was found for their corresponding ligands (**L1** and **L3**) with a higher quantum yield (Figure S15d, Table S1). However, the emission from complex **2** and ligand **L2** was too weak to detect.

### X‐ray crystallography

**Figure 2:**
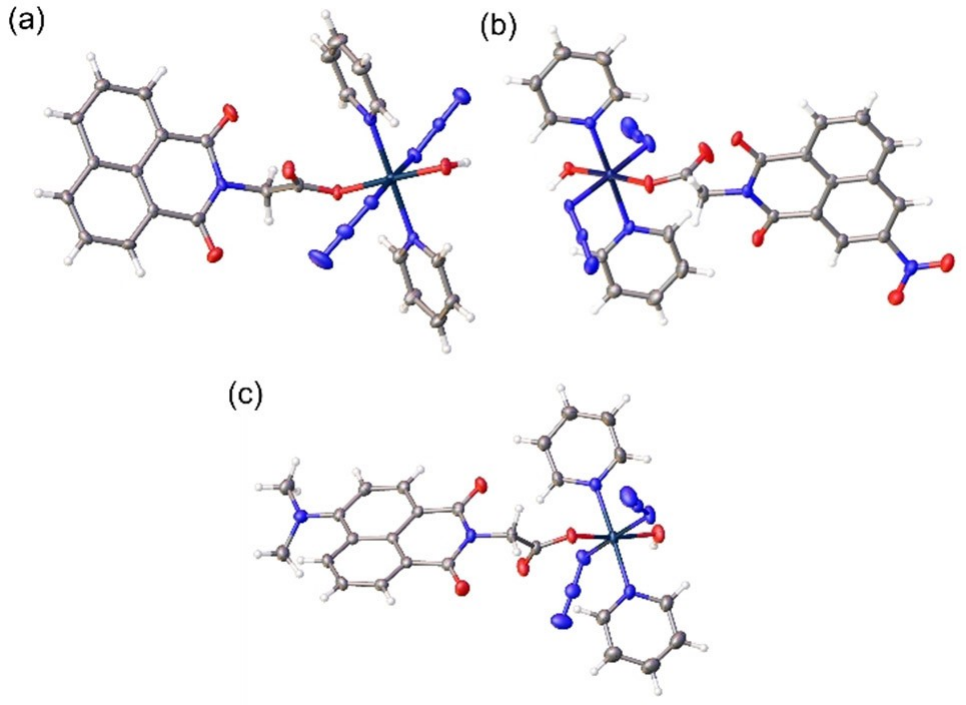
X‐ray crystal structures of octahedral Pt^IV^ complexes a) **1**, b) **2**, and c) **3**, with thermal ellipsoids drawn at the 50 % probability level.

Crystals suitable for X‐ray diffraction studies of complexes **1**–**3** were obtained through the diffusion of ethers into solutions in DMSO/DMF for **1** and DCM/MeOH for **2** and **3**. In the crystal structures of **1**–**3**, the Pt centres with slightly distorted octahedral geometry resemble those of reported photoactive Pt^IV^ complexes with the O−Pt−O angles less than 180° (**1** (177.17(7)°), **2** (175.53(10)°), and **3** (170.01(9)°), Figure 2, Tables S2 and S3). The naphthalimide rings in **1** form infinite π‐stacks along the *a* axis of the cell with the distance between closest antiparallel naphthalimide rings being 3.516 Å. The large planar structure of the naphthalimide ring indicates the possibility of DNA intercalation. Due to the π stacking and H‐bonds (O1−H1⋅⋅⋅N3), an infinite chain is formed with all adjacent molecules being antiparallel to each other (Table S4). The substituents cause slight distortions of the naphthalimide ring in **2** and **3**, which reduces their ability to π‐stack. Hydrogen bonds between the axial O−H and O in the carbonyl group close to Pt in an adjacent molecule (O100−H100⋅⋅⋅O15) in complex **2**, and between an axial O−H and terminal azide N of an adjacent molecule (O1−H1⋅⋅⋅N3) in complex **3** are observed.

### DFT calculations of absorption and fluorescence spectra

The crystal structure‐based DFT‐calculated UV/vis spectra exhibit absorption bands comparable with experimental spectra (Figure S16). Two main types of absorbing states are observed in the predicted absorption spectra. The transitions responsible for the band at approximately 300 nm correspond to ligand‐to‐Pt^IV^ charge‐transfer (LMCT) states, transferring electronic density from the azide π system to an antibonding orbital centred on Pt^IV^ (302 and 308 nm for **1**, 301, 303, 306 and 309 nm for **2**, and 301 and 308 nm for **3**). The antibonding characters of these transitions are either directed towards the azide ligand, or against the hydroxide and naphthalimide ligands, which resembles the calculation for **FM‐190**, and are responsible for the photo‐release of azide, hydroxido and naphthalimide ligands due to the antibonding character of lowest unoccupied natural transition orbitals.^[3d]^ The transitions of **1** located at 347 nm correspond to the naphthalimide π‐to‐π* excitation. NO_2_ and NMe_2_ substituents, respectively, blue‐ and red‐shift naphthalimide absorption from 347 nm in **1** to 334 nm in **2**, and 436 nm in **3**. In addition, two other transitions with partial LMCT character were observed for **3** at 256 and 257 nm, corresponding to π‐donation from the naphthalimide ligand to antibonding orbitals between Pt^IV^ and pyridines, hydroxido and naphthalimide ligands. The computed emission wavelengths of the free protonated ligand released from **1** and **3** are *λ*_max_=387 nm and *λ*_max_=497 nm, respectively. They are both blue‐shifted compared to experimental measurements, but support emission from the photo‐released ligands.

### Photoactivation and dark stability

**Figure 3:**
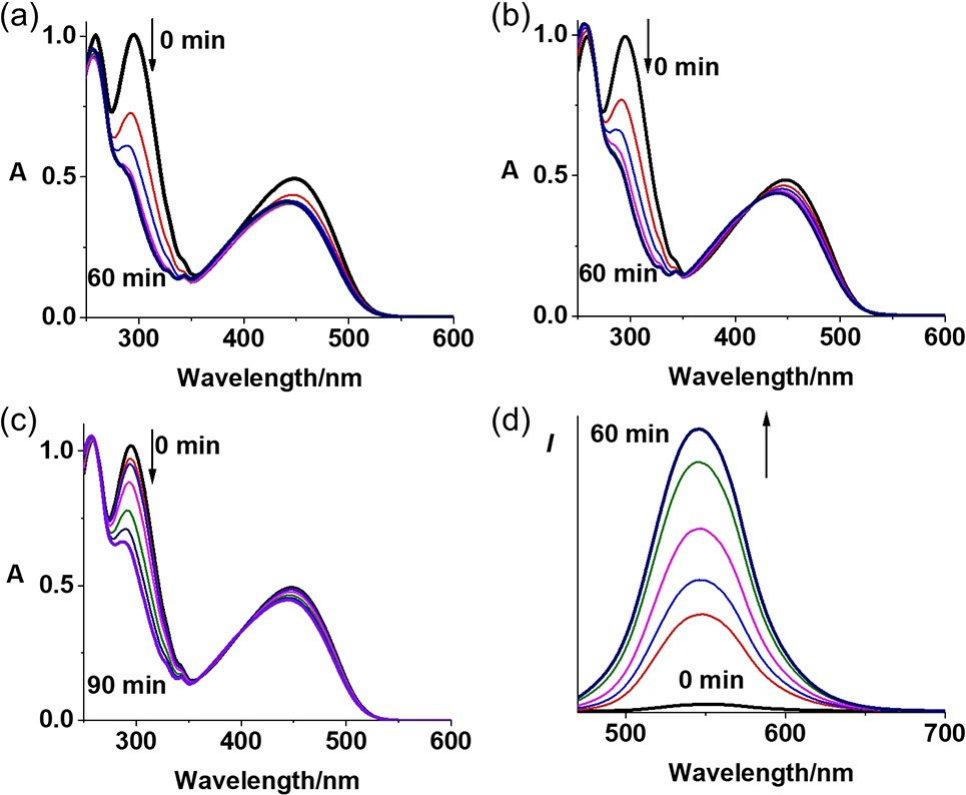
UV/vis spectral changes for **3** in RPMI‐1640 cell culture medium upon irradiation with a) indigo 420 nm, b) blue 463 nm, and c) green 517 nm light. d) Fluorescence change of **3** in PBS upon irradiation with blue light (463 nm).

Complexes **1**–**3** are stable in DMSO for 2 h in the dark (Figure S17). Complex **3** also shows high dark stability in RPMI‐1640, and in the presence of GSH (2.4 mM, Figures S17b and S18). The photo‐decomposition of **1**–**3** was monitored by UV/vis and fluorescence spectroscopy after various time intervals and irradiation with indigo (420 nm), blue (463 nm) or green (517 nm, for **3** only) light at 298 K (Figures 3, S19 and S20). A rapid decrease in the LMCT (N_3_→Pt) bands of **1**–**3** at around 300 nm, was observed upon irradiation, indicating the release of azide ligands. The absorbance of **3** at 450 nm decreased and blue‐shifted after irradiation; this suggests the release of the naphthalimide ligand. The emission of **1** and **3** increases gradually with irradiation (463 nm), indicating the release of the axial naphthalimide ligand (Figures 3d and S20). As neither complex **2** nor the free nitro‐naphthalimide ligand **L2** shows any apparent fluorescence, no change in emission was detected for **2**.

Upon irradiation of **3** with indigo light (420 nm), the LC‐MS peak for **3** decreased gradually and disappeared within 30 min, accompanied by the formation of Pt^IV^ species {Pt^IV^(py)_2_(N_3_)(OH)(gly‐4‐NMe_2_‐Nap)}^+^ (708.81 *m*/*z*, b), Pt^II^ species {Pt^II^(py)_2_(CH_3_CN)(N_3_)}^+^ (436.00 *m*/*z*, a) and released axial ligand gly‐4‐NMe_2_‐Nap (+H, 298.48 *m*/*z*, c; Figure S21). About 66 % of **3** decomposed after 60 min irradiation with green light (517 nm, Figure S21b). In contrast, **FM‐190** exhibited negligible decomposition after similar irradiation with green light (Figure S22).

### Photo‐reaction with 5’‐GMP (guanosine 5’‐monophosphate)

The interaction between photoactivated **3** and 5’‐GMP as a model nucleotide was investigated using LC–MS. An aqueous solution of **3** and 5’‐GMP was irradiated with indigo (420 nm) or green (517 nm) light at 298 K, then analysed by LC–MS (Figure S23). Major Pt^II^‐GMP adducts were detected as {Pt^II^(py)_2_(CH_3_CN)(GMP−H)}^+^ (755.73 *m*/*z*, G1) and {Pt^II^(py)_2_(N_3_)(GMP)}^+^ (757.74 *m*/*z*, G2). Indigo light induced the formation of more adducts than green light after the same irradiation time.

### DNA binding calculations and experimental studies in cell‐free media

Electrostatic maps (ESMs) of iso‐density surface (Figure S24) show that all complexes exhibit a cavity‐like region of negative electrostatic potential, produced by the spatial proximity of the N_3_ ligand, the carbonyl of the gly carboxylate and the nearby naphthalimide carbonyls. The minimum of electrostatic potential (ESP) for all complexes is in the cavity, with minima of −0.100, −0.090, and −0.106 au, for complexes **1**, **2**, and **3**, respectively. This nucleophilic region could be responsible for the initial step of the intercalation process in these complexes. Accordingly, the ability to attract DNA bases through electrostatic interactions toward the naphthalimide ligand in these complexes can be ranked as **3**>**1**>**2**. The naphthalimide ligand can then stabilise the intercalation between bases due to π‐stacking. However, complex **3** may not give rise to proper π‐stacking because of its bulky NMe_2_ ligand at the 4 position.

To investigate the binding of complexes **1**–**3** to double‐stranded calf thymus DNA in the dark, LD spectra of DNA in the absence or presence of Pt complexes were recorded in 10 mM Tris‐Cl, pH 7.4 containing 0.77–2.3 *v*/*v* DMSO. Complex **1** and, to a lesser extent **2**, induced a negative LD signal in the 300–500 nm region, where the complexes exhibit weak absorption (Figure S25). Thus, the existence of LD signals in this region gives evidence that **1** and **2** interact with double‐stranded *ct*‐DNA and become oriented in flow. Moreover, the interaction of **1** and **2** with DNA gives rise to LD signals of the same sign as DNA itself (i. e., negative). This result shows that the chromophores of **1** and **2** are oriented parallel with DNA base pairs, which is indicative of intercalative binding. In contrast, **3** did not show any induced LD bands, despite its higher absorbance in the visible region. This suggests that **3** does not bind to DNA, or its binding affinity is very low. However, all three complexes may interfere with the DNA band in the 260 nm region due to their own absorptivity. The presence of DMSO can also contribute slightly to DNA structural changes (Figure S25). Hence the changes in the LD band around 260 nm cannot be interpreted reliably.

**Figure 4:**
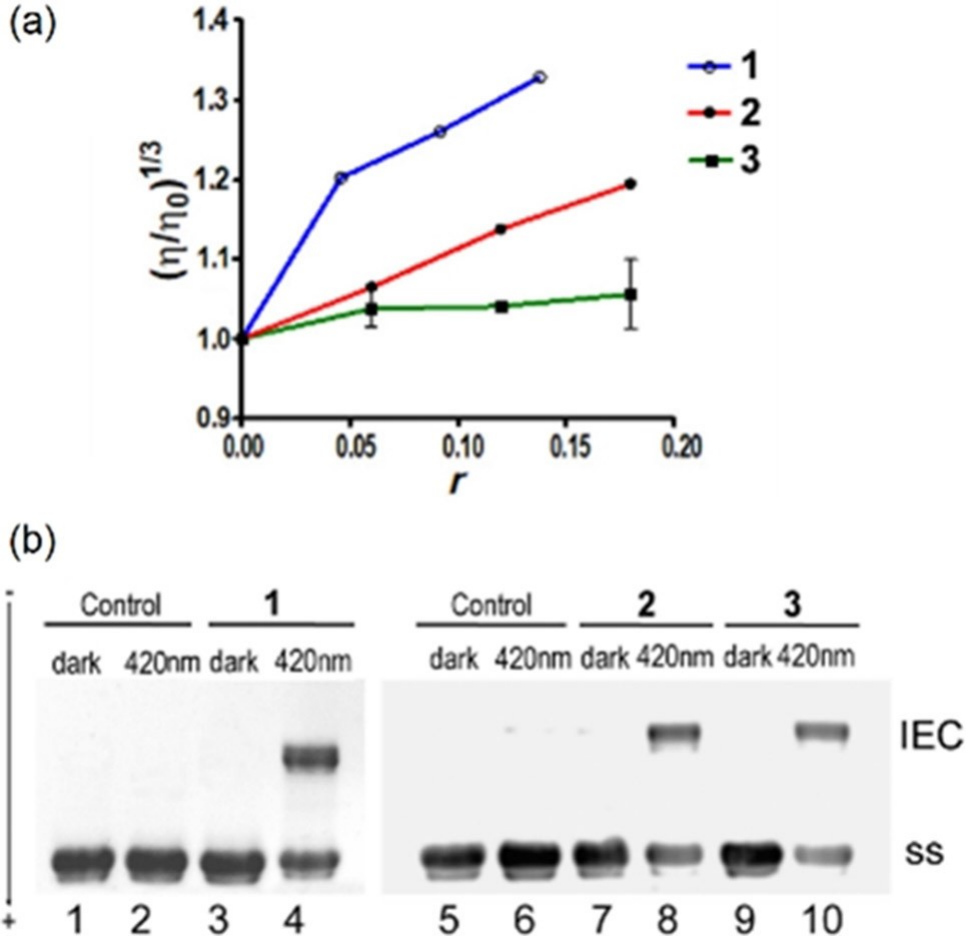
a) The relative specific viscosity of calf thymus DNA in the presence of complexes **1**, **2** or **3** in the dark. Data are presented as (*η*/*η*_0_)^1/3^ vs. *r* ([Pt]/[DNA]), where *η* is the viscosity of DNA in the presence of Pt complex and *η*_0_ is the viscosity of DNA alone in the buffer containing the same percentage of DMSO as the respective sample. b) Agarose gel electrophoresis for determination of DNA interstrand crosslinking. The interstrand crosslinks (IEC) were formed by **1**, **2**, or **3** under irradiation conditions (420 nm light) in linear pSP73 plasmid DNA. Lanes 1, 2, 5, and 6: control, unmodified DNA; lanes 3 and 4: DNA modified by **1** at *r*=0.0009; lanes 7 and 8: DNA modified by **2** at *r*=0.0012; lanes 9 and 10: DNA modified by **3** at *r*=0.0012.

To characterise the DNA binding mode in the dark further, viscometry measurements were performed (Figure 4a). Complexes **1** and **2** increase the length of DNA, resulting in an increased viscosity, **2** being less effective than **1**. In contrast, **3** does not lengthen the DNA helix, as no increase in the viscosity was observed. These data correspond to LD results and confirm intercalative DNA binding of **1** and, to a lesser extent, **2**. However, either no, or extremely weak DNA binding was confirmed for complex **3**, ascribed to its bulky 4‐NMe_2_ substituent.

To verify that complexes **1**–**3** can bind strongly to DNA under irradiation, we analysed linearised plasmid DNA modified by **1**–**3** by gel electrophoresis under denaturing conditions (Figure 4b). With 1 h exposure (420 nm), a significant fraction of slowly migrating DNA was observed, which can be attributed to interstrand crosslinking by irradiated Pt compounds, but no crosslinked fraction was observed for these samples when kept in the dark. The intensity of the interstrand crosslink‐containing fraction was concentration‐dependent (Figure S26). Notably, complexes **1** and **2** displayed *ca*. 2x higher interstrand crosslinking fractions compared with **3**, which may result from their pre‐intercalation in the dark, shortening the distance between Pt and DNA bases thus facilitating the formation of strong crosslinks (Figure S26d).

### Photo‐cytotoxicity and cellular accumulation studies

Due to the poor aqueous solubility of **1**, the dark‐photo‐cytotoxicity of only complexes **2** and **3** was determined in human A2780 ovarian, A549 lung and PC3 prostate cancer cells, and MRC5 lung normal cells using the sulforhodamine B (SRB) colorimetric assay (Table 1). Satisfactory dark stability with IC_50_ values >50 μM was observed for **2**, and >100 μM for **3** in cells. However, **2** and **3** exhibited significant photo‐cytotoxicity upon irradiation (465 nm, 4.8 mW cm^−2^, 1 h) with IC_50_ values of 1.2–13.5 μM. Pre‐intercalation of the NO_2_‐Nap naphthalimide ligand of **2** might contribute to the enhanced photo‐cytotoxicity of **2**. Notably, photo‐cytotoxicity with green light (520 nm, 11.7 mW cm^−2^, 1 h) was detected for complex **3** (IC_50_=32.7–92.8 μM), which can be attributed to its photoactivation with green light. No cytotoxicity of **L2** and **L3** ligands alone was observed regardless of the presence of irradiation due to the short incubation time, indicating the important role of Pt^IV^ fragments in killing cancer cells.

**Table 1:** IC_50_ values and photo‐cytotoxicity indices (PI) for complexes **2** and **3** obtained after 1 h of incubation, 1 h of irradiation (465/520 nm) and 24 h of recovery for human A549 lung, PC3 prostate, A2780 ovarian cancer cells, and MRC5 normal fibroblasts. Data for complex **FM‐190** and ligands **L2** and **L3** are listed for comparison.

Complex	Irradiation	IC_50_ [μM]^[a]^/PI			
		A549	PC3	A2780	MRC5
**2**	dark	>50	>50		
	465 nm	11.8±0.1	6.4±0.7		
	PI blue	>4.2	>7.5		
**3**	dark	>100	>100	>100	>100
	465 nm	13.5±1.3	10.1±0.2	1.2±0.1	
	520 nm	92.8±13.5	32.7±0.5	35.3±2.2	
	PI blue	>7.4	>9.8	>82.6	
	PI green	>1.0	>3.0	>2.8	
**FM‐190** ^[13]^	dark	>100	>100	>100	>100
	465 nm	51.9±2.5	55.6±0.9	7.1±0.4	
	520 nm	>100	>100	>100	
	PI blue	>1.9	>1.7	>14.0	
**L2**	dark	>100	>100		
	465 nm	>100	>100		
**L3**	dark	>100	>100		
	465 nm	>100	>100

[a] All data are from two independent experiments.

Pt accumulation from complexes **2** and **3** (10 μM dose) in A2780 ovarian, A549 lung and PC3 prostate cancer cells in the dark was at least 15x higher than from **FM‐190** (Table S5). Notably, approximately 3x enhanced Pt accumulation for complex **2** over **3** was observed; this is consistent with its higher photo‐cytotoxicity. The high lipophilicity of **2** and **3** probably contributes to their high cellular accumulation.

### Photo‐oxidation of NADH

The coenzyme nicotinamide adenine dinucleotide (NADH) plays an important role in maintaining the redox balance in cancer cells and is a potential target of photooxidation.^[14]^ A solution of NADH (2 mM) was irradiated with indigo light (420 nm) in the presence of complex **3**, then analysed by LC‐MS (Figure S27). The decrease in intensity of the peak for NADH (665.61 *m*/*z*, NADH+H^+^) and the appearance of a new peak for NAD^+^ (663.62 *m*/*z*) were observed after 1 h irradiation. The amount of NAD^+^ produced by **3** is about 1.9x as much as that produced by **FM‐190**. Little NAD^+^ was detected after irradiation of NADH alone. The results indicate the ability of these Pt^IV^ complexes to induce photo‐oxidation of biomolecules, giving rise to a combined mechanism of action of DNA binding and ROS generation.

### ROS generation

**Figure 5:**
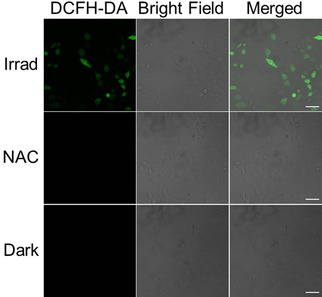
Confocal fluorescence microscopy images of ROS generation in A549 cells treated with complex **3** (IC_50_ concentration, 2 h in the dark, or 1 h in the dark and 1 h of irradiation, 465 nm) then probed by DCFH‐DA (20 μM, *λ*_ex_=488 nm, *λ*_em_=507–611 nm) in the absence and presence of antioxidant *N*‐acetyl‐l‐cysteine (NAC, 10 mM). Scale bars: 50 μm.

Photo‐induced singlet oxygen was detected for complex **3** in acetonitrile directly by infrared fluorescence spectroscopy (Figure S28). The cellular ROS generation of **3** was determined by the DCFH‐DA assay (Figure 5). A549 lung cancer cells were treated with **3** for 1 h at 310 K in the dark at 13.5 μM (photo IC_50_ concentration). One dish was exposed to 1 h irradiation (465 nm), while the other was kept in the dark. Cells were stained with DCFH‐DA (20 μM) then analysed using confocal microscopy.

Clear green fluorescence (*λ*_ex_=488 nm, *λ*_em_=507–611 nm) in irradiated cells indicates the generation of ROS (Figure 5). In contrast, cells kept in the dark or treated with antioxidant *N*‐acetyl cysteine (NAC, 10 mM) exhibited no apparent fluorescence under the same conditions.

## Conclusions

We have synthesised and characterised three novel photoactive diazido Pt^IV^‐naphthalimide complexes **1**–**3** that are dark‐stable, even in the presence of bio‐reductant GSH. Their X‐ray crystal structures highlight the large π surface of the naphthalimide. DFT calculations assigned the LMCT transitions with antibonding character, favourable for the photo‐release of azide, hydroxide and naphthalimide ligands. Complexes **1**–**3** undergo photo‐decomposition upon irradiation with visible light (420 and 463 nm). Photoactivated complexes can form DNA crosslinks by binding to guanine bases. Complexes **1** and **2** pre‐intercalate into DNA in the dark, facilitating the formation of covalent crosslinks, and thus displayed approximately twice as many interstrand crosslinks as **3**. In addition, complex **2** displayed approximately three times as much cellular accumulation as that of **3**, contributing to enhanced photo‐cytotoxicity. Photo‐induced cellular ROS production contributes to their photo‐cytotoxicity. Notably, a 4‐NMe_2_ substituent induced red‐shifted absorption of the naphthalimide ring to 450 nm allowing photo‐decomposition of **3** with green light (517 nm), giving rise to its green‐light photo‐cytotoxicity. The introduction of versatile naphthalimide ligands into photoactive diazido Pt^IV^ is a promising strategy to improve photo‐cytotoxicity, capable of providing pre‐irradiation intercalative DNA recognition and red‐shifting activation wavelengths, which might facilitate their preclinical development.

## Conflict of interest

The authors declare no conflict of interest.

## Supporting information

As a service to our authors and readers, this journal provides supporting information supplied by the authors. Such materials are peer reviewed and may be re‐organized for online delivery, but are not copy‐edited or typeset. Technical support issues arising from supporting information (other than missing files) should be addressed to the authors.

Supporting Information
