# Supplementary material for: DNA‐Intercalative Platinum Anticancer Complexes Photoactivated by Visible Light
Source: Chemistry. 2021 May 27;27(41):10711–6. doi: 10.1002/chem.202101168 (PMC8361943; doi:10.1002/chem.202101168)
Supplement: Supplementary file 1 — Supporting Information [file CHEM-27-10711-s001.pdf]

Chemistry–A European Journal

Supporting Information

DNA-Intercalative Platinum Anticancer Complexes Photoactivated by Visible Light

Huayun Shi, Jana Kasparkova, Clément Soulié, Guy J. Clarkson, Cinzia Imberti, Olga Novakova, Martin J. Paterson, Viktor Brabec, and Peter J. Sadler*

Experimental section 1
Table S1. Photophysical properties of complexes **1**–**3** and ligands **L1**–**L3** in PBS. 9
Table S2. Crystal data and structure refinement for **1**–**3**. 9
Table S3. Selected bond lengths (Å) and bond angles (°) for **1**–**3**. 10
Table S4. Selected hydrogen bonds parameters for **1**–**3**. 10
Figure S1. HPLC purity of Pt(IV) complexes with a 1,8-naphthalimide ligand (**1**–**3**). 11
Figure S2. 400 Hz ^{1}H NMR spectrum of complex **1** in DMSO–d_6 at 298 K. 12
Figure S3. 100 Hz DEPT-135 ^{13}C NMR spectrum of complex **1** in DMSO–d_6 at 298 K. 12
Figure S4. 400 Hz ^{1}H NMR spectrum of complex **2** in DMSO–d_6 at 298 K. 13
Figure S5. 125 Hz DEPT-135 ^{13}C NMR spectrum of complex **2** in DMSO–d_6 at 298 K. 13
Figure S6. 400 Hz ^{1}H NMR spectrum of complex **3** in $CDCl_3$ at 298 K. 14
Figure S7. 125 Hz DEPT-135 ^{13}C NMR of complex **3** in $CDCl_3$ at 298 K. 14
Figure S8. Chemical structures of 1,8-naphthalimide ligands. 15
Figure S9. 400 Hz ^{1}H NMR spectrum of ligand **L1** in DMSO–d_6 at 298 K. 15
Figure S10. 125 Hz DEPT-135 ^{13}C NMR spectrum of ligand **L1** in DMSO–d_6 at 298 K. 16
Figure S11. 400 Hz ^{1}H NMR spectrum of ligand **L2** in DMSO–d_6 at 298 K. 16
Figure S12. 125 Hz DEPT-135 ^{13}C NMR spectrum of ligand **L2** in DMSO–d_6 at 298 K. 17
Figure S13. 400 Hz ^{1}H NMR spectrum of ligand **L3** in $CDCl_3$ at 298 K. 17
Figure S14. 125 Hz DEPT-135 ^{13}C NMR spectrum of ligand **L3** in $CDCl_3$ at 298 K. 18
Figure S15. (a) UV-vis absorption spectra and (b) fluorescence spectra of complexes **1**–**3** and ligands **L1**–**L3** (c and d) in PBS with 5% DMSO at 298 K. 18
Figure S16. DFT-predicted absorption spectra of **1** (a), **2** (b), and **3** (c) with superposed histograms of the transition oscillator strengths. 19
Figure S17. Dark stability of **1**–**3** in DMSO; and **3** in phenol red-free RPMI-1640. 20
Figure S18. Dark stability of **3** in the presence of GSH. 20
Figure S19. UV-vis absorption spectra showing photochemical decomposition of **1** and **2**. 21
Figure S20. Fluorescence changes during photodecomposition of **1**. 21
Figure S21. Photochemical decomposition of **3** determined by HPLC.. 22
Figure S22. Photochemical decomposition of **FM-190** determined by HPLC. 22
Figure S23. Photoreaction between of **3** and 5'-GMP monitored by HPLC. 23
Figure S24. Electrostatic potential mapped on isodensity (n=0.001) surfaces for complexes **1**–**3**. 23
Figure S25. LD spectra of calf thymus DNA in the absence (**—**) or presence of complexes **1**–**3**. 24
Figure S26. Denaturing agarose gel electrophoresis for determination of DNA interstrand cross-linking by **1**–**3** and quantitative evaluation of the IEC (interstrand cross-links) fraction of the irradiated samples. 24
Figure S27. Photooxidation of NADH by complexes monitored by HPLC. 25
Figure S28. Infrared phosphorescence detection of $^{1}O_2$ formation by **3**. 25
References 26

Experimental section

Materials and Instruments. *O*-(Benzotriazol-1-yl)-*N*, *N*, *N'*, *N'*-tetramethyluronium tetrafluoroborate (TBTU) was purchased from Merck, Pyridine was from Fischer Scientific UK, K_2PtCl_4, NaN_3, H_2O_2 (30%), Fmoc-gly-OH, *N*, *N*-diisopropylethylamine (DIPEA), piperdine, 1,8-naphthalic anhydride, 4-bromo-1,8-naphthalic anhydride, 3-nitro-1,8-naphthalic anhydride and other chemicals were from Sigma Aldrich and used without further purification. 4-Dimethylamino-1,8-naphthalic anhydride,[S1] and *trans*, *trans*, *trans*-[Pt(py)$_2$(N$_3$)$_2$(OH)$_2$] (**FM-190**)[S2] were prepared according to reported methods.

NMR spectra were recorded on Bruker Avance III 400 MHz (for ^{1}H) or Bruker Avance III HD 500 MHz (for ^{1}H) spectrometers with the residual signal of the solvent used as a reference. ESI-MS spectra were recorded on an Agilent 6130B single quadrupole detector instrument and ESI-HR-MS data were collected on a Bruker microTOF instrument for positive ions at 298 K.

Electronic absorption spectra were recorded on a Varian Cary 300 UV-vis spectrophotometer in a 1 cm quartz cuvette and solvent used as reference. Jasco FP-6500 Spectrofluorometer was used to record fluorescence spectra.

Analytical reversed-phase HPLC analyses were carried out on an Agilent ZORBAX Eclipse XDB-C18 column (250×4.6 mm, 5 µm, flow rate: 1 mL/min), using linear gradients of 0.1% formic acid in H_2O (solvent A) and 0.1% formic acid in CH_3CN (solvent B, 10-80% in 30 min). LC-MS was carried out on Bruker Amazon X mass spectrometry connected online with an Agilent 1260 HPLC.

The light sources used for photoactivation were an LZC-ICH2 photoreactor (Luzchem Research Inc.) equipped with a temperature controller and 8 Luzchem LZC-420 lamps without light filtration, and LED light sources (BASETech model no. SP-GU10 230 V~50 Hz 1.3-2.1 W) with λ_{max} = 463 or 517 nm. A 96-array of LEDs with λ_{max} = 465 (4.8 mW cm^{-2} per LED) or 520 (11.7 mW cm^{-2} per LED) nm was used for *in vitro* growth inhibition.

Platinum content was analysed on an ICP-MS 7500cx (Agilent) or ICP-OES 5300DV (Perkin Elmer). The emission wavelength detected for Pt in ICP-OES was 265.945 nm and ^{195}Pt was determined in ICP-MS with ^{166}Er (50 ppb) as an internal standard.

Synthesis and characterisation. *Caution!* Although we encountered no problem during the work reported here, due care and attention with appropriate precautions should be taken in the synthesis and handling of heavy metal azides since they can be shock-sensitive. All work involved metal azides was carried out in the dark.

***Trans, trans, trans*-[Pt(py)$_2$(N$_3$)$_2$(OH)(gly)]** (**Pt-gly-NH$_2$**). To the solution of complex **FM-190** (50.0 mg, 106 µmol), Fmoc-gly-OH (31.6 mg, 106 µmol), and TBTU (34.2 mg, 106 µmol) in DMF (3 mL), DIPEA (100 µL) was added. The reaction mixture was stirred overnight at 298 K under a nitrogen atmosphere. After evaporation to dryness, the oily residue was collected and purified by column chromatography on silica gel (2% methanol + 98% DCM) to give ***trans, trans, trans*-[Pt(py)$_2$(N$_3$)$_2$(OH)(gly-Fmoc)]** (**Pt-gly-Fmoc**) (ESI-MS: $[M + Na]^+$ (*m/z*) Calc., 773.2; Found, 773.3). The yellow residue was re-dissolved in piperdine/DMF (20:80) and stirred overnight at 298 K under a nitrogen atmosphere. After evaporation to dryness, the oily residue was dissolved in a small amount of DCM and a precipitate was formed immediately upon addition of excess diethyl ether. The solid was collected and used directly in the next step of the synthesis without further purification. ^{1}H NMR (CDCl$_3$, 400 MHz): 8.92 (d with Pt satellites, J = 6.2 Hz, J ^{195}Pt-^{1}H = 26.8 Hz, 4H, H_α *py*), 8.26 (t, J = 7.5 Hz, 2H, H_γ *py*), 7.79 (t, J = 6.4 Hz, 4H, H_β *py*), 3.74 (s, 2H, CH_2), 3.36 (s, 1H, *OH*), 3.32 (s, 2H, NH_2). ESI-MS: $[M + H]^+$ (*m/z*) Calc., 529.1; Found, 529.3.

***Trans, trans, trans*-[Pt(py)$_2$(N$_3$)$_2$(OH)(gly-Nap)]** (**1**). *Trans, trans, trans*-[Pt(py)$_2$(N$_3$)$_2$(OH)(gly)] (**Pt-gly-NH$_2$**, 20.0 mg, 38 µmol) and 1,8-naphthalic anhydride (7.5 mg, 38 µmol) were suspended in DMF and the reaction mixture was stirred overnight at 353 K under a nitrogen atmosphere. After evaporation to dryness, the oily residue was collected and purified by column chromatography on silica gel (4% methanol + 96% DCM). Yield: 25.1%. ^{1}H NMR (DMSO-d_6, 400 MHz): 8.75 (d with Pt satellites, J = 6.0 Hz, J ^{195}Pt-^{1}H = 25.8 Hz, 4H, H_α *py*), 8.54 (d, J = 7.1 Hz, 2H, *Hna1*), 8.47 (d, J = 8.1 Hz, 2H, *Hna3*), 8.24 (t, J = 7.0 Hz, 2H, H_γ *py*), 7.88 (d, J = 7.7 Hz, 2H, *Hna2*), 7.76 (t, J = 6.9 Hz, 4H, H_β *py*), 4.74 (s, 2H, CH_2), 3.40 (s, 1H, *OH*). ^{13}C NMR (DMSO-d_6, 100 MHz): 169.69 (*COO*), 163.69 (*C(O)NC(O)*), 149.63 (C_α *py*), 142.51 (C_γ *py*), 135.00 (*Cna*), 131.80 (*Cna*), 131.35 (*Cna*), 127.85 (*Cna*), 127.68 (*Cna*), 126.62 (C_β *py*), 122.31 (*Cna*), 43.46 (CH_2). ESI-HR-MS: $[M + Na]^+$ (*m/z*) Calc., 731.1051; Found, 731.1043.

***Trans, trans, trans*-[Pt(py)$_2$(N$_3$)$_2$(OH)(gly-3-NO$_2$-Nap)]** (**2**). *Trans, trans, trans*-[Pt(py)$_2$(N$_3$)$_2$(OH)(gly)] (**Pt-gly-NH$_2$**, 40.0 mg, 76 µmol) and 3-nitro-1,8-naphthalic anhydride (21 mg, 76 µmol) were suspended in ethanol and the reaction mixture was stirred overnight at 353 K under a nitrogen atmosphere. After evaporation to dryness, the oily residue was collected

and purified by column chromatography on silica gel (5% methanol + 95% DCM). Yield: 13.3%. ^{1}H NMR (DMSO-d_6, 400 MHz): 9.52 (s, 1H, *Hna2*), 8.99 (s, 1H, *Hna1*), 8.82-8.72 (m, 6H, *Hna3+5* and H_α *py*), 8.26 (t, *J* = 7.4 Hz, 2H, H_γ *py*), 8.08 (t, *J* = 8.0 Hz, 1H, *Hna4*), 7.80 (t, *J* = 5.9 Hz, 4H, H_β *py*), 4.75 (s, 2H, CH_2), 4.0 (s, 1H, *OH*). ^{13}C NMR (DMSO-d_6, 125 MHz): 170.32 (*COO*), 163.02 (*C(O)NC(O)*), 162.51 (*C(O)NC(O)*), 149.64 (C_α *py*), 146.43 (*Cna*), 142.62 (C_γ *py*), 137.14 (*Cna*), 134.66 (*Cna*), 131.40 (*Cna*), 130.54 (*Cna*), 129.95 (*Cna*), 129.79 (*Cna*), 126.75 (C_β *py*), 124.10 (*Cna*), 123.64 (*Cna*), 122.72 (*Cna*), 43.74 (CH_2). HR-MS: $[M + H]^+$ (*m/z*) Calc., 754.1082; Found, 754.1079.

***Trans, trans, trans*-[Pt(py)$_2$(N$_3$)$_2$(OH)(gly-4-NMe$_2$-Nap)]** (**3**). *Trans, trans, trans*-[Pt(py)$_2$(N$_3$)$_2$(OH)(gly)] (**Pt-gly-NH$_2$**, 33.0 mg, 63 μmol) and 4-dimethylamino-1,8-naphthalic anhydride (16.7 mg, 69 μmol) were suspended in ethanol and the reaction mixture was stirred overnight at 353 K under a nitrogen atmosphere. After evaporation to dryness, the oily residue was collected and purified by column chromatography on silica gel (3% methanol + 97% DCM). Yield: 13%. ^{1}H NMR (CDCl$_3$, 400 MHz): 8.91 (d with Pt satellite, *J* = 5.9 Hz, *J* ^{195}Pt-^{1}H = 26.5 Hz, 4H, H_α *py*), 8.55 (d, *J* = 7.2 Hz, 1H, *Hna3*), 8.46 (d, *J* = 8.2 Hz, 1H, *Hna1*), 8.42 (d, *J* = 8.5 Hz, 1H, *Hna5*), 8.01 (t, *J* = 7.6 Hz, 2H, H_γ *py*), 7.64 (t, *J* = 7.6 Hz, 1H, *Hna4*), 7.59 (t, *J* = 6.8 Hz, 4H, H_β *py*), 7.10 (d, *J* = 8.2 Hz, 1H, *Hna2*), 4.95 (s, 2H, CH_2), 3.48 (s, 1H, *OH*), 3.10 (s, 6H, $N(CH_3)_2$). ^{13}C NMR (CDCl$_3$, 125 MHz): 171.68 (*COO*), 164.47 (*C(O)NC(O)*), 163.87 (*C(O)NC(O)*), 157.0 (*Cna*), 149.70 (C_α *py*), 141.09 (C_γ *py*), 132.59 (*Cna*), 131.22 (*Cna*), 130.96 (*Cna*), 130.50 (*Cna*), 125.97 (C_β *py*), 125.35 (*Cna*), 124.78 (*Cna*), 123.19 (*Cna*), 115.13 (*Cna*), 113.24 (*Cna*), 44.80 (CH_3), 43.30 (CH_2). ESI-HR-MS: $[M + Na]^+$ (*m/z*) Calc., 774.1460; Found, 774.1463.

Synthesis of ligands. Ligands **L1–L3** were prepared for comparison using modified methods reported.[S4-S6] To the solution of glycine methyl ester hydrochloride (1.1 mmol) and 1,8-naphthalic anhydride (or its derivatives, 1 mmol) in DMF (3 mL), triethylamine (1 mmol) was added. The mixture was stirred overnight at 373 K. After evaporation to dryness, the solid was collected and purified by column chromatography on silica gel (DCM). The solid was suspended in the mixture of methanol and water (5:4) containing 4 mol. equiv. NaOH, stirred at 343 K for 5 h. The solution was acidified with HCl.

L1. A white precipitate was collected and washed with DCM. ^{1}H NMR (DMSO, 400 MHz): 13.19 (s, 1H, *COOH*), 8.57-8.52 (m, 4H), 7.92 (t, *J* = 7.8 Hz, 2H), 4.75 (s, 2H, CH_2). ^{13}C NMR (DMSO, 125 MHz): 169.82, 163.59, 135.36, 131.87, 131.62, 127.87, 121.99, 41.64. ESI-MS: $[M - H]^-$ (*m/z*) Calc., 254.0; Found, 254.0.

L2. A yellow precipitate was collected and washed with DCM. ^{1}H NMR (DMSO, 400 MHz): 13.19 (s, 1H, *COOH*), 9.54 (s, 1H), 8.99 (s, 1H), 8.84 (d, *J* = 8.2 Hz, 1H), 8.72 (d, *J* = 7.0 Hz, 1H), 8.10 (t, *J* = 7.7 Hz, 1H), 4.77 (s, 2H, CH_2). ^{13}C NMR (DMSO, 125 MHz): 169.52, 162.91, 162.44, 146.42, 137.38, 134.84, 131.47, 130.75, 129.92, 129.87, 123.92, 123.70, 122.36, 41.88. ESI-MS: $[M - H]^-$ (*m/z*) Calc., 299.0; Found, 299.0.

L3. The resulting solution was washed with DCM, then evaporated to dryness. The solid was dissolved in DCM and the remaining solid was filtered off to give an orange solid after drying. ^{1}H NMR ($CDCl_3$, 400 MHz): 8.61 (d, *J* = 7.3 Hz), 8.52 (d, *J* = 8.3 Hz), 8.49 (d, *J* = 8.8 Hz), 7.69 (t, *J* = 7.8 Hz), 7.15 (d, *J* = 8.2 Hz), 5.00 (s, 2H), 3.15 (s, 6H). ^{13}C NMR ($CDCl_3$, 125 MHz): 171.55, 164.27, 163.61, 157.43, 133.21, 131.77, 131.54, 130.57, 125.28, 124.88, 122.48, 114.15, 113.29, 44.78, 40.77. ESI-MS: $[M - H]^-$ (*m/z*) Calc., 297.1; Found, 297.1.

X-Ray crystallography. Single crystals of **1** were grown from DMSO+DMF/Et_2O and those of **2** and **3** were grown from DCM+MeOH/Et_2O. A suitable crystal was selected and mounted on a glass fibre with Fomblin oil and placed on a Rigaku Oxford Diffraction SuperNova diffractometer with a duel source (Cu at zero) equipped with an AtlasS2 CCD area detector. The crystal was kept at 150(2) K during data collection. Using Olex2[S7], the structure was solved with the ShelXT[S8] structure solution program using Intrinsic Phasing and refined with the ShelXL[S9] refinement package using Least Squares minimisation.

X-ray crystallographic data for complexes **1–3** have been deposited in the Cambridge Crystallographic Data Centre under the accession numbers CCDC 2054009, 2054011 and 2054010, respectively. X-ray crystallographic data in CIF format are available from the Cambridge Crystallographic Data Centre (http://www.ccdc.cam.ac.uk/).

Dark stability and photodecomposition. Complexes (A_{300nm} = 1) in RPMI-1640 without phenol red or DMSO kept in the dark were monitored by UV-vis to test their dark stability at 298 K. Complex **3** (30 μM) was also mixed with 2.4 mM GSH in aqueous solution and

monitored by LC-MS. The photodecomposition of complex **1–3** in RPMI-1640 without phenol red was monitored by UV-vis spectroscopy, that in PBS was monitored by fluorescence spectroscopy, and that in aqueous solution was monitored by LC-MS at different time intervals after irradiation with indigo (420 nm), blue (463 nm), and green (517 nm) light at 298K.

DFT calculations

DFT calculations were performed with Gaussian 16, version A.03 using the B3LYP functional and GD3BJ long-range correction. Geometry optimization were performed with the 6-311g** basis set for H, N, C, O, and SDD effective core potential (ECP60 and electron basis) for Pt, starting from X-ray data. Frequency calculations on the resulting geometries showed no imaginary frequency. The absorption spectrum of each complex optimized geometry was investigated with TD-DFT calculations (convoluted over 60 states), using a 6-311+G** basis set for H, N, C, O, and SDD (ECP60 and electron basis) for Pt. All calculations were done with water as implicit solvent using the Conductor-like Polarisation Continuum Model (CPCM). ESP maps were plotted on 0.001 electronic density iso-surface.

Photooxidation of NADH. An aqueous solution with 60 μM Pt(IV) complex and 2 mM NADH was irradiated with indigo light (420 nm) for 1 h, then analysed by LC-MS immediately.

Photoreaction with 5'-GMP. 30 μM complex was mixed with 2 mol. equiv. of guanosine 5′-monophosphate disodium salt hydrate (5′-GMP-Na_2) in aqueous solution. The solution was irradiated for 1 h (420/517 nm) and analysed immediately on a Bruker Amazon X mass spectrometer connected online with the HPLC.

LD spectroscopy. LD spectra of calf thymus DNA (3×10^{-4} M) in the absence or presence of Pt(IV) complexes were recorded with a Jasco J-720 spectropolarimeter using a flow Couette cell consisting of a fixed outer cylinder and a rotating solid quartz inner cylinder, separated by a gap of 0.5 mm, giving a total path length of 1 mm. The spectra represent the mean of two recordings from 600 to 220 nm, at a scan rate of 500 nm/min, using a 0.5 nm step, 2 nm width, and 0.25 s averaging time. All spectra were obtained from samples in 10 mM Tris-Cl, pH 7.4 at room temperature.

Viscometry. Viscosity was measured using an AMVn Automated MicroViscometer (Anton Paar GmbH, Austria) in a 10 mM M Tris–HCl buffer (pH 7.4) at 310 K using a 1.6 mm capillary tube. A mean of six replicated measurements were taken for accuracy. The concentration of calf thymus DNA was kept constant (0.46×10^{-4} M), while the concentration of Pt(IV) complexes varied as indicated. The data were plotted as $(\eta/\eta_0)^{1/3}$ versus [Pt]/[DNA] ratio, where η is the specific viscosity of DNA in the presence of Pt(IV) complex and η_0 is the viscosity of free DNA in the buffer containing the same percentage of DMSO as respective sample with Pt complex.

DNA interstrand cross-linking. DNA cross-linking ability was investigated as described previously with small modifications.[S10] Briefly, plasmid pSP73 DNA (2464 bp) was linearized by EcoRI endonuclease. The resulting linear fragment of DNA was purified by phenol/chloroform extraction and precipitation, and 3´-end-labeled by the Klenow fragment of DNA polymerase I and [α-^{32}P]dATP. The DNA was then mixed with Pt(IV) complex, and the mixture was divided into two portions. One aliquot was then irradiated (1 h, 420 nm, 310 K) and subsequently incubated for an additional 18 h in the dark at 310 K. The other aliquot was kept in the dark for 19 h at 310 K. The samples were then analysed by electrophoresis using a 1% agarose gel under denaturing conditions. After the electrophoresis was completed, the gels were dried, visualized using a GE Healthcare FLA 7000 bioimager. To estimate the fractions of non-cross-linked and cross-linked DNA in each lane, the intensity of the individual bands was assessed by using AIDA image analyser software.

Cell culture. Human cell lines, ovarian carcinoma A2780, lung adenocarcinoma A549, prostate cancer PC3 cells and lung healthy MRC-5 cells were obtained from the European Collection of Animal Cell Culture (ECACC), Salisbury, UK. All cell lines used in this work were grown in Roswell Park Memorial Institute media (RPMI-1640), which was supplemented with 10% v/v of foetal calf serum (FCS) and 1% v/v penicillin/streptomycin. The adherent monolayers of cells were grown at 310 K in a humidified atmosphere containing 5% CO_2 and passaged regularly at *ca*. 80% confluence.

Photo-dark cytotoxicity. Approximate 1.5 × 10^4 cells were seeded per well in 96-well plates. Independent duplicate plates were used, one for dark, the other for irradiation experiments. The cells were pre-incubated in drug-free medium with phenol red at 310 K for 24 h. Complexes were dissolved first in DMSO and then diluted in phenol red-free RPMI-1640 to make the stock solution of the drug. These stock solutions were further diluted using phenol-red free cell culture medium until working concentrations were achieved, the maximum DMSO concentration was < 0.5% v/v in these solutions. Cells were exposed to the complexes at different concentrations for 1 h. Then one plate was irradiated for 1 h using blue light (4.8 mW cm^{-2} per LED at 465 nm) or green light (11.7 mW cm^{-2} per LED at 520 nm), while the dark plate was kept in the incubator. After irradiation, supernatants of both plates were removed by suction and the cells were washed with phosphate-buffered saline (PBS). Photocytotoxicity was determined after another 24 h recovery at 310 K in drug-free phenol red-containing medium by comparison to untreated controls which were only exposed to vehicle. Untreated controls were also compared between the irradiated and the non-irradiated plates to ensure that the differences in cell survival were not statistically relevant, hence guaranteeing that the differences in cell viability observed were not due to the light source. The SRB assay was used to determine cell viability.[S11] Absorbance measurements of the solubilised dye (on a Promega microplate reader) allowed the determination of viable treated cells compared to untreated controls. IC_{50} values (concentrations which caused 50% of cell death) were determined as the average of triplicates and their standard deviations were calculated. Stock concentrations for all metal complexes used in these biological assays were adjusted/verified after ICP-OES metal quantification.

Platinum accumulation in cancer cells in the dark. For Pt cellular accumulation studies, *ca*. 5×10^6 A2780, A549 or PC3 cells were plated in 100 mm Petri dishes and allowed to attach for 24 h, then the plates were exposed to complexes at 10 μM. Additional plates were incubated with medium alone as a negative control. After 1 h of incubation in the dark at 310 K, the cells were rinsed three times with cold PBS and harvested by trypsinisation. The number of cells in each sample was counted manually using a haemocytometer. Then the cells were centrifuged to obtain the whole cell pellet for ICP-MS analysis. All experiments were conducted in triplicate. The whole cell pellets were dissolved in concentrated 72% v/v nitric acid (200 μL), then transferred into Wheaton v-vials (Sigma-Aldrich) and heated in an oven at 343 K

overnight. Each cellular sample solution was diluted with Milli-Q water (3.8 mL), to obtain a final HNO_3 concentration of *ca*. 3.6% v/v.

Singlet oxygen ($^1O_2$) detection. Infrared phosphorescence of Pt(IV) complexes was measured on a Fluorolog-3 spectrofluorometer (Jobin Yvon Horiba, Model FL3-11) with a 450 W Xenon lamp light source and a solid state liquid nitrogen cooled Indium Gallium Arsenide detector. The excitation light power at 420/450 nm is *ca*. 1 mW. A 850 nm longpass glass filter from Thorlabs (FGL850S) was used between the light source and detector.

Intracellular ROS generation determined by confocal fluorescence microscopy. Fluorescence images were recorded on a confocal microscope (LSM 880, AxioObserver). A549 cells (*ca*. 1×10^5) were seeded in glass-bottom cell culture dishes (CELLview) and cultured for 24 h to allow attachment. Cells were exposed to complex (13.5 μM) in the absence and presence of NAC for 1 h in dark, then 1 h irradiated with blue light (465 nm). Supernatant was removed and cells were incubated with DCFH-DA (20 μM) for 40 min. Cells were washed with HBSS before measurement. Cells treated with complex (13.5 μM) in the dark were used for comparison. The fluorescence images were recorded on a fluorescence confocal microscope (LSM 880, AxioObserver, $\lambda_{ex}/\lambda_{em}$ = 488/507–611 nm).

Table S1. Photophysical properties for complexes **1–3** and ligands **L1–L3** in PBS.

Complex	λ_{abs}/nm (ε_{max}/dm^{3}mol^{-1}cm^{-1})	λ_{em}/nm	Φ_{em}[a]
**1**	259 (35906), 306 (52683), 341 (45322), 355 (sh, 36167)	381, 395 (max)	0.002
**2**	267 (44199), 279 (46099), 293 (43349), 335 (sh, 20616)	[b]	[b]
**3**	260 (29900), 296 (28743), 450 (14095)	546 (max)	0.006
**L1**	342 (28653)	380, 395 (max)	0.03
**L2**	278 (33646), 335 (14359)	[b]	[b]
**L3**	258 (18950), 284 (13407), 327 (3898), 344 (3714), 444 (13613)	549	0.013

[a] $Ru(bpy)_3Cl_2$ in air-saturated H_2O used as a reference (λ_{ex} = 450).[S12] [b] Complex **2** and ligand **L2** are not fluorescent.

Table S2. Crystal data and structure refinement for complexes **1–3**.

Complex	**1**	**2**	**3**
CDDC number	2054009	2054011	2054010
Empirical formula	$C_{24}H_{19}N_9O_5Pt$	$C_{24}H_{18}N_{10}O_7Pt$	$C_{26}H_{24}N_{10}O_5Pt$
Formula weight	708.57	753.57	751.64
Temperature/K	150(2)	150(2)	150(2)
Crystal system	triclinic	monoclinic	monoclinic
Space group	P-1	$P2_1/n$	I2/a
a/Å	7.40510(12)	8.6623(3)	15.6369(3)
b/Å	8.24493(17)	22.7102(5)	7.99463(15)
c/Å	21.2074(4)	13.1536(4)	42.5857(7)
α/°	92.7868(16)	90	90
β/°	90.0429(14)	104.584(3)	95.8821(17)
γ/°	106.8637(16)	90	90
Volume/Å^3	1237.51(4)	2504.25(13)	5295.67(18)
Z	2	4	8
ρ_{calc}g/cm^3	1.902	1.999	1.886
μ/mm^{-1}	5.725	5.672	5.359
F(000)	688.0	1464.0	2944.0
Crystal size/mm^3	0.32 × 0.12 × 0.06 light yellow block	0.4 × 0.18 × 0.1	0.35 × 0.2 × 0.06 yellow block
Radiation	MoKα (λ = 0.71073)	MoKα (λ = 0.71073)	MoKα (λ = 0.71073)
2Θ range for data collection/°	5.17 to 60.054	4.808 to 59.838	5.186 to 60.814
Index ranges	$-10 \le h \le 10$, $-11 \le k \le 11$, $-28 \le l \le 29$	$-11 \le h \le 11$, $-31 \le k \le 31$, $-16 \le l \le 17$	$-21 \le h \le 19$, $-11 \le k \le 10$, $-58 \le l \le 60$
Reflections collected	59521	30619	33072

Independent reflections	6820 [R_{int} = 0.0512, R_{sigma} = 0.0322]	6673 [R_{int} = 0.0363, R_{sigma} = 0.0333]	7190 [R_{int} = 0.0340, R_{sigma} = 0.0307]
Data/restraints/parameters	6820/84/480	6673/272/512	7190/0/382
Goodness-of-fit on F^2	1.035	1.191	1.085
Final R indexes [$I>=2\sigma(I)$]	R_1 = 0.0223, wR_2 = 0.0424	R_1 = 0.0326, wR_2 = 0.0569	R_1 = 0.0274, wR_2 = 0.0573
Final R indexes [all data]	R_1 = 0.0265, wR_2 = 0.0439	R_1 = 0.0390, wR_2 = 0.0584	R_1 = 0.0389, wR_2 = 0.0618
Largest diff. peak/hole / e Å^{-3}	1.20/-1.13	1.05/-0.95	1.22/-1.18

Table S3. Selected bond lengths (Å) and bond angles (°) for complexes **1**–**3**.

**1**		**2**		**3**	
Pt1–N1	2.060(2)	Pt1–N201	2.056(3)	Pt1–N1	2.051(3)
Pt1–N4	2.042(2)	Pt1–N401	2.038(3)	Pt1–N4	2.049(3)
Pt1–N7	2.035(2)	Pt1–N101	2.051(3)	Pt1–N7	2.046(3)
Pt1–N13	2.0372(19)	Pt1–N301	2.034(3)	Pt1–N13	2.027(3)
Pt1–O1	1.9814(16)	Pt1–O100	1.968(2)	Pt1–O1	1.974(2)
Pt1–O19	2.0333(16)	Pt1–O16	2.054(3)	Pt1–O19	2.051(2)
N1–N2	1.210(3)	N101–N102	1.208(5)	N1–N2	1.201(4)
N2–N3	1.145(3)	N102–N103	1.146(5)	N2–N3	1.139(4)
N4–N5	1.219(3)	N201–N22A	1.215(8)	N4–N5	1.222(4)
N5–N6	1.145(3)	N22A–N23A	1.155(10)	N5–N6	1.133(4)
O1–Pt1–O19	177.17(7)	O100–Pt1–O16	175.53(10)	O1–Pt1–O19	170.01(9)
C20–O19–Pt1	123.32(14)	C15–O16–Pt1	131.4(2)	C20–O19–Pt1	126.29(18)
O19–C20–C21	109.8(5)	O15–C15–C14	122.0(3)	O19–C20–C21	111.3(3)
N22–C21–C20	111.1(8)	N2–C14–C15	113.6(19)	N22–C21–C20	111.5(2)
C23–N22–C34	126.6(13)	C3–N2–C1	124(3)	C23–N22–C22	125.2(2)

Table S4. Selected hydrogen bond parameters for complexes **1**–**3**.

	**D**	**H**	**A**	**d(D-H)/Å**	**d(H-A)/Å**	**d(D-A)/Å**	**D-H-A/°**
**1**	O1	H1	N3^1	0.84	2.31	3.039(3)	145.4
**2**	O100	H100	O15^2	0.84	2.03	2.863(4)	171.9
**3**	O1	H1	N3^3	0.84	2.24	3.012(4)	153.6

^{1}1-X,-Y,-Z; ^{2}-0.5+X,0.5-Y,-0.5+Z; ^{3}+X,-1+Y,+Z

Table S5. Accumulation of Pt (ng/10^6 cells) in cancer cells after exposure to complexes **2** or **3** (10 μM, 1 h, in dark). Data for **FM-190** are listed for comparison.[S13]

Complex	Platinum accumulation (ng/10^6 cells)[a]		
	A549	PC3	A2780
**2**	68.3±15.2[*]	73.3±13.4[*]	ND
**3**	20.7±1.3[**]	23.2±1.4[*]	18.4±3.5[*]
**FM-190**	1.0±0.2[*]	1.5±0.3[*]	1.19±0.04[***]

[a] All data are from triplicate samples and their statistical significance evaluated by a two-tail t-test with unequal variances. [*]$p < 0.05$, [**]$p < 0.01$, [***]$p < 0.005$. ND = not determined.

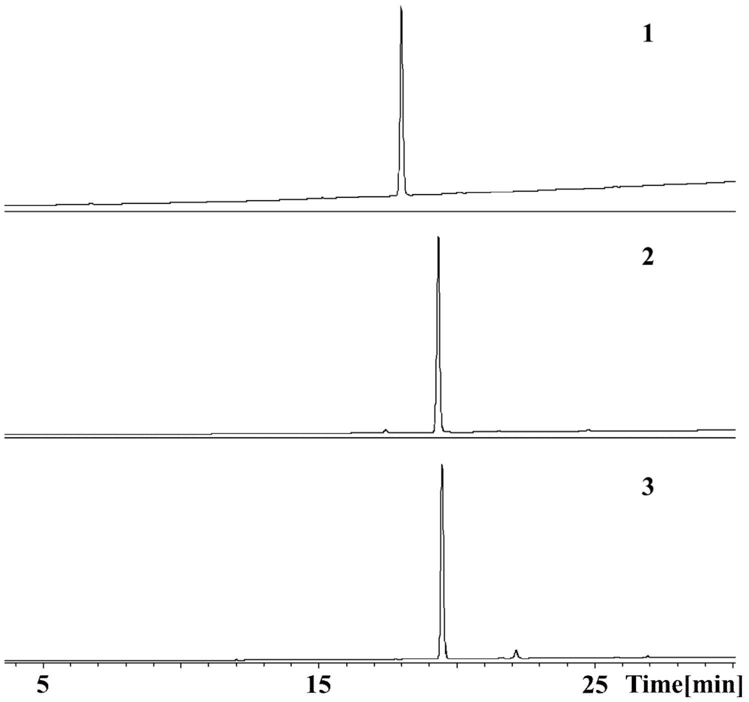

Figure S1. HPLC purity of Pt(IV) complexes with a 1,8-naphthalimide ligand (**1**–**3**), gradient 10–80% CH_3CN in 30 min, detection wavelength 254 nm.

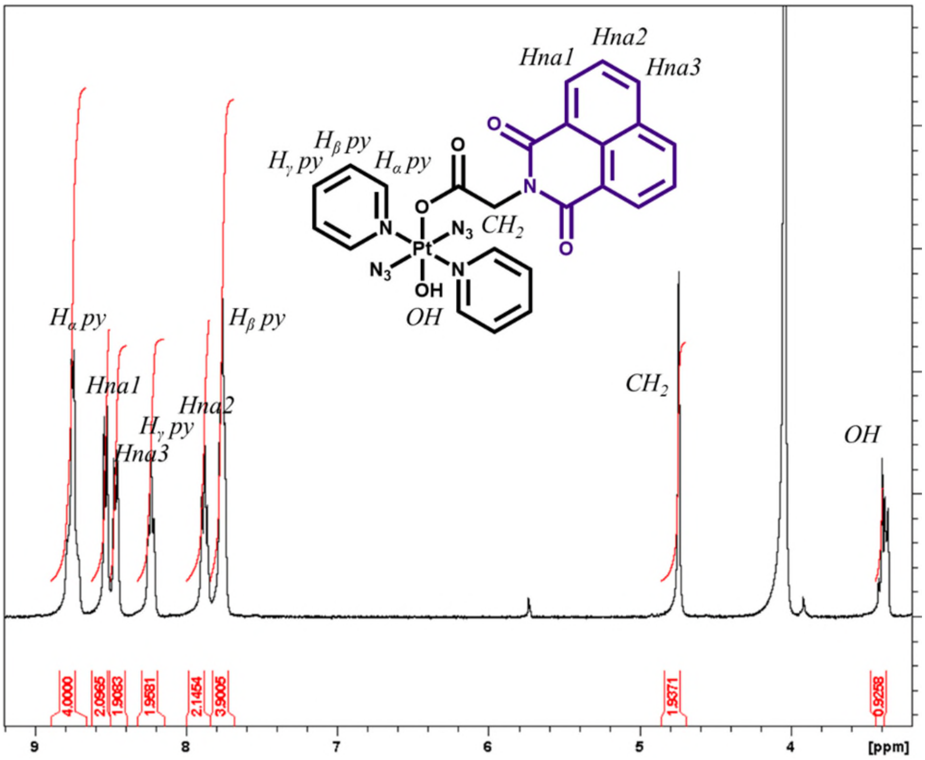

Figure S2. 400 Hz ^{1}H NMR spectrum of complex **1** in DMSO–d_6 at 298 K.

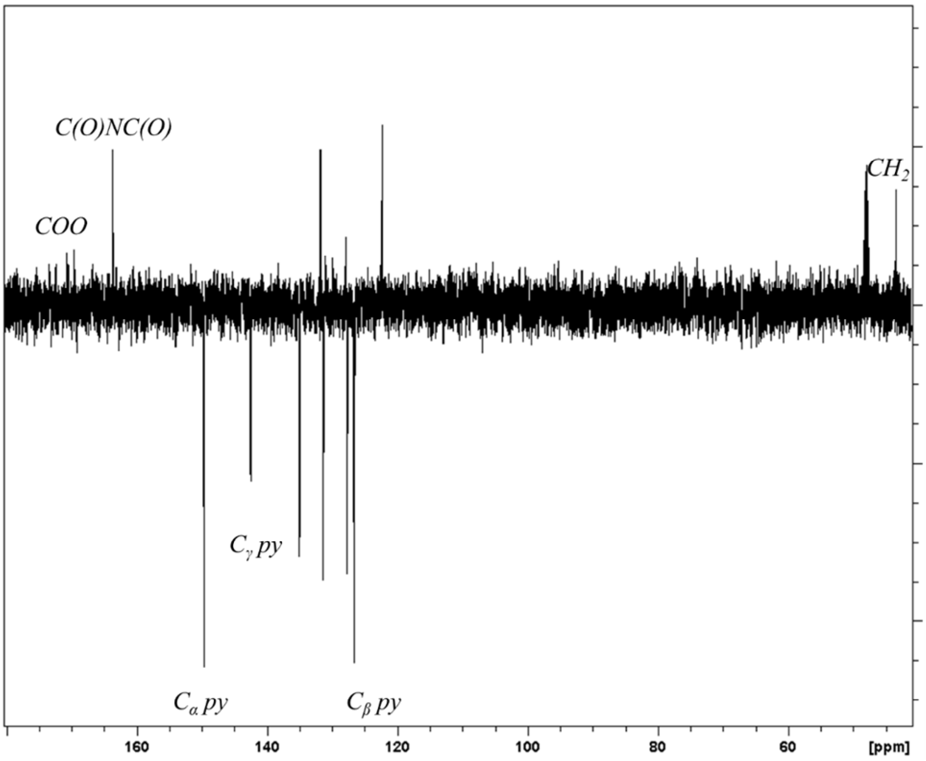

Figure S3. 100 Hz DEPT-135 ^{13}C NMR spectrum of complex **1** in DMSO–d_6 at 298 K.

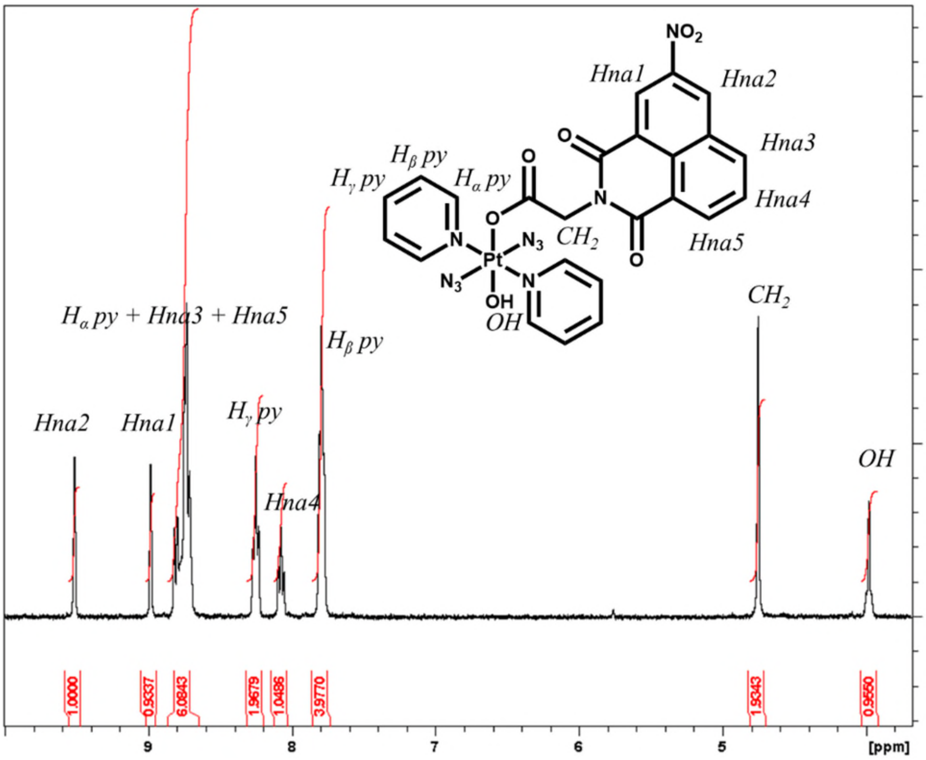

Figure S4. 400 Hz ^{1}H NMR spectrum of complex **2** in DMSO–d_6 at 298 K.

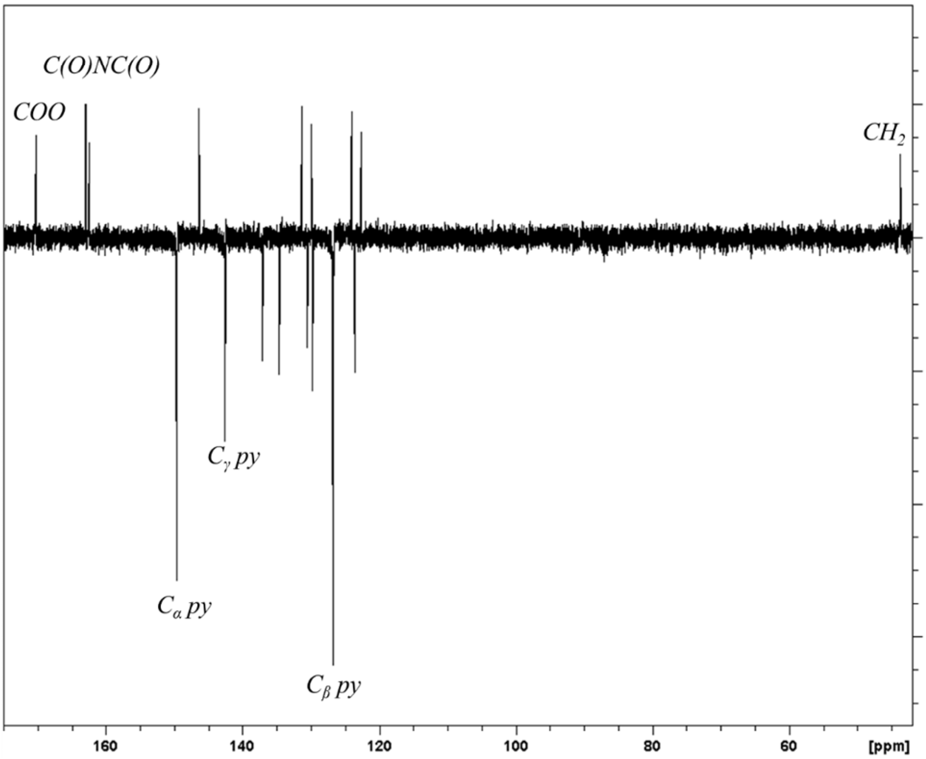

Figure S5. 125 Hz DEPT-135 ^{13}C NMR spectrum of complex **2** in DMSO–d_6 at 298 K.

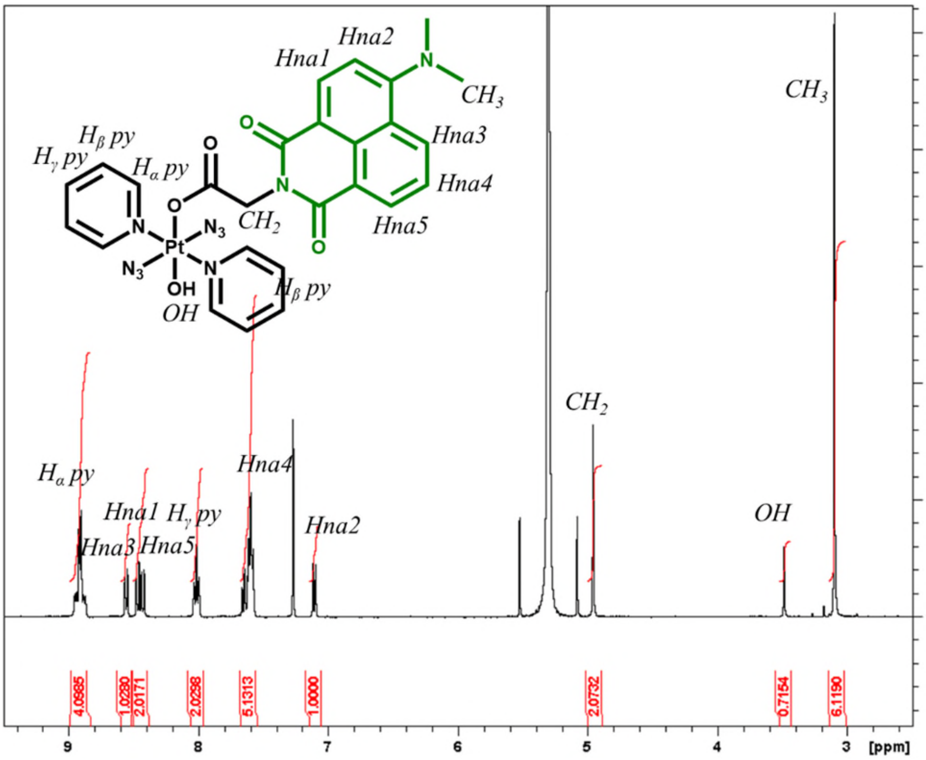

Figure S6. 400 Hz ^{1}H NMR spectrum of complex **3** in $CDCl_3$ at 298 K.

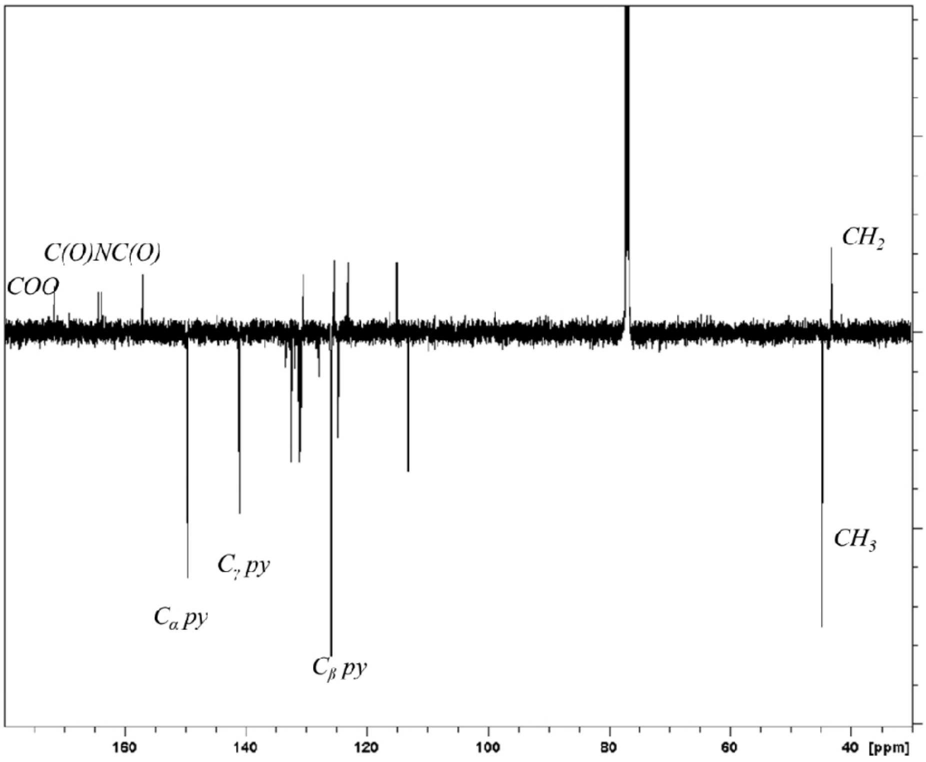

Figure S7. 125 Hz DEPT-135 ^{13}C NMR of complex **3** in $CDCl_3$ at 298 K.

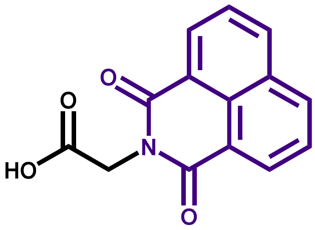

L1

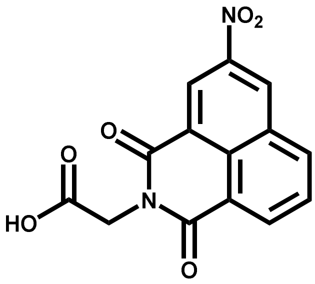

L2

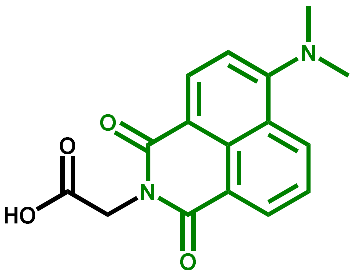

L3

Figure S8. Chemical structures of 1,8-naphthalimide ligands H-gly-R-Nap, R = H (**L1**), 3-NO_2 (**L2**) or 4-NMe_2 (**L3**).

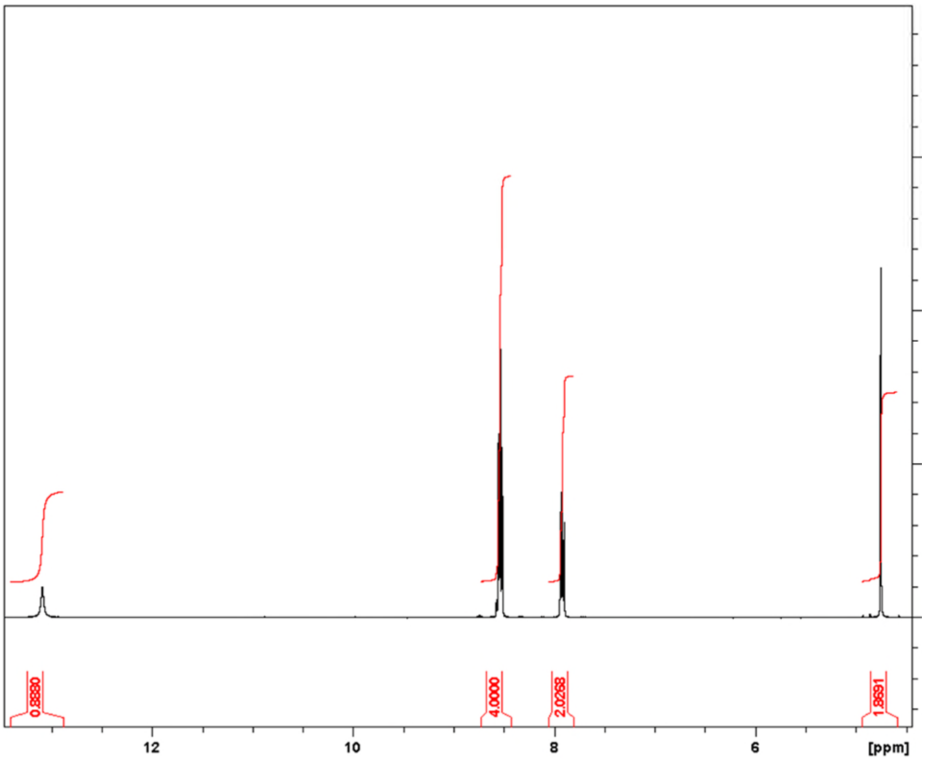

Figure S9. 400 Hz ^{1}H NMR spectrum of ligand **L1** in DMSO–d_6 at 298 K.

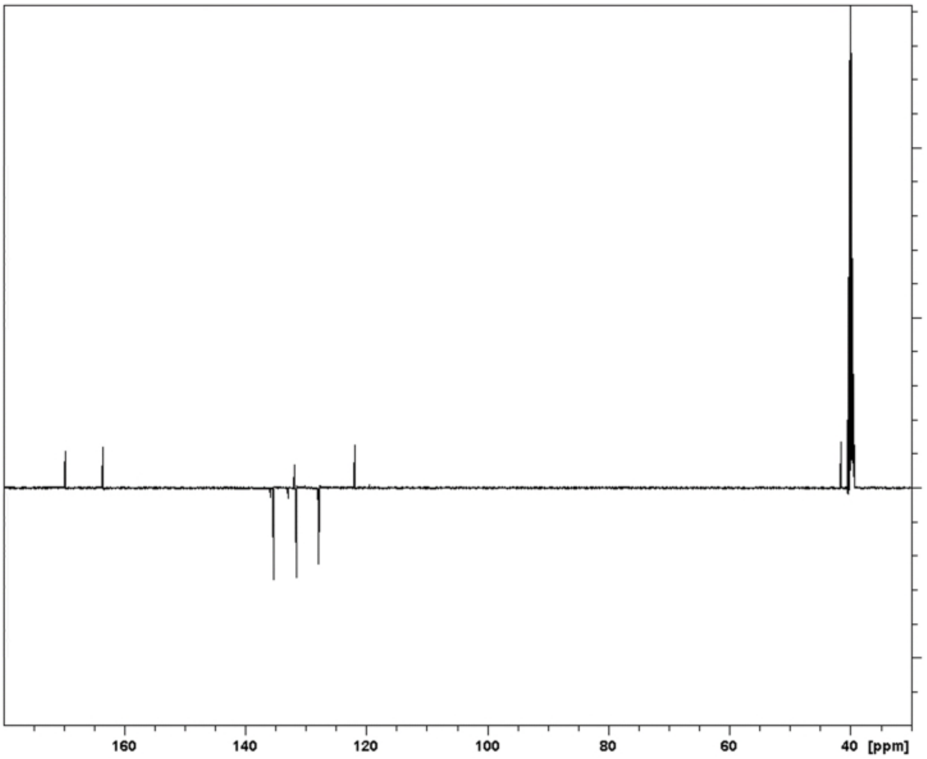

Figure S10. 125 Hz DEPT-135 ^{13}C NMR spectrum of ligand **L1** in DMSO–d_6 at 298 K.

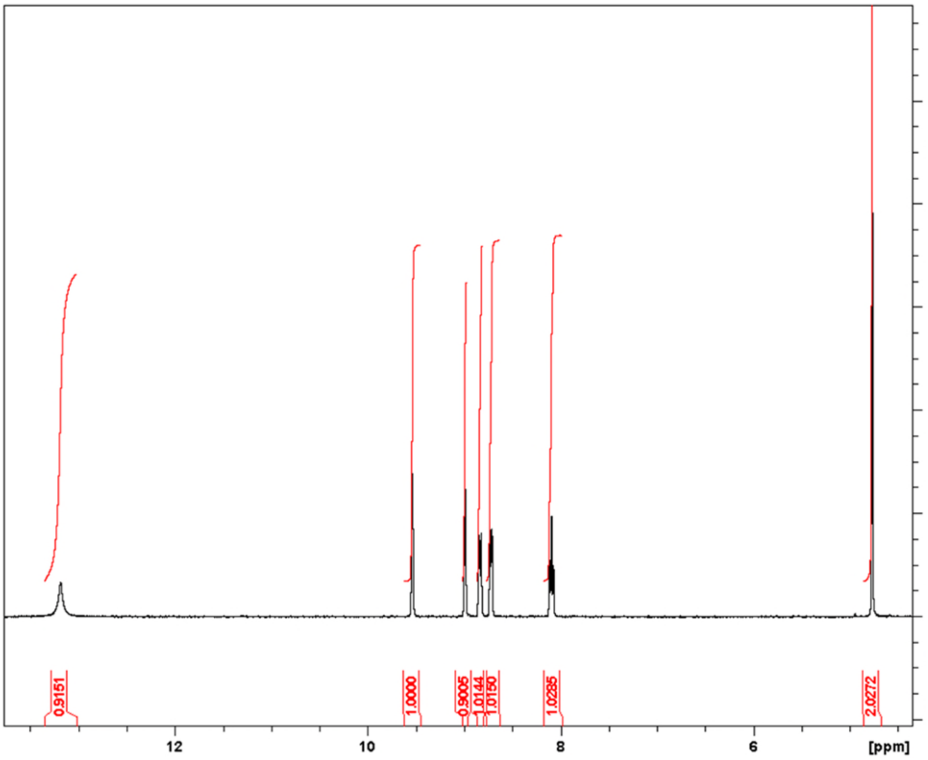

Figure S11. 400 Hz ^{1}H NMR spectrum of ligand **L2** in DMSO–d_6 at 298 K.

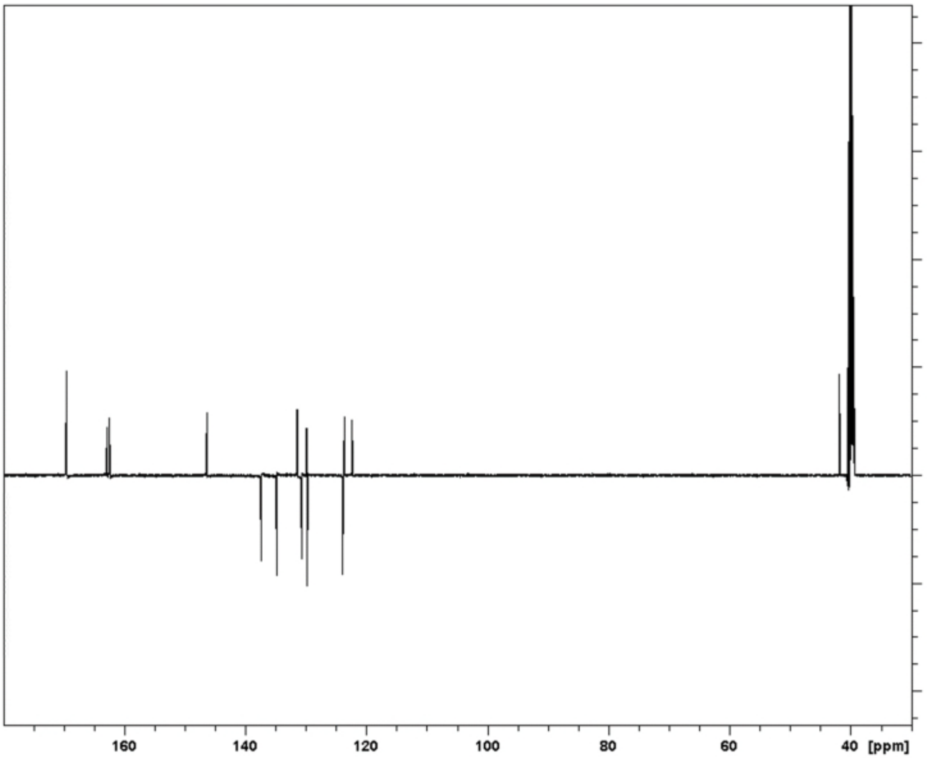

Figure S12. 125 Hz DEPT-135 ^{13}C NMR spectrum of ligand **L2** in DMSO–d_6 at 298 K.

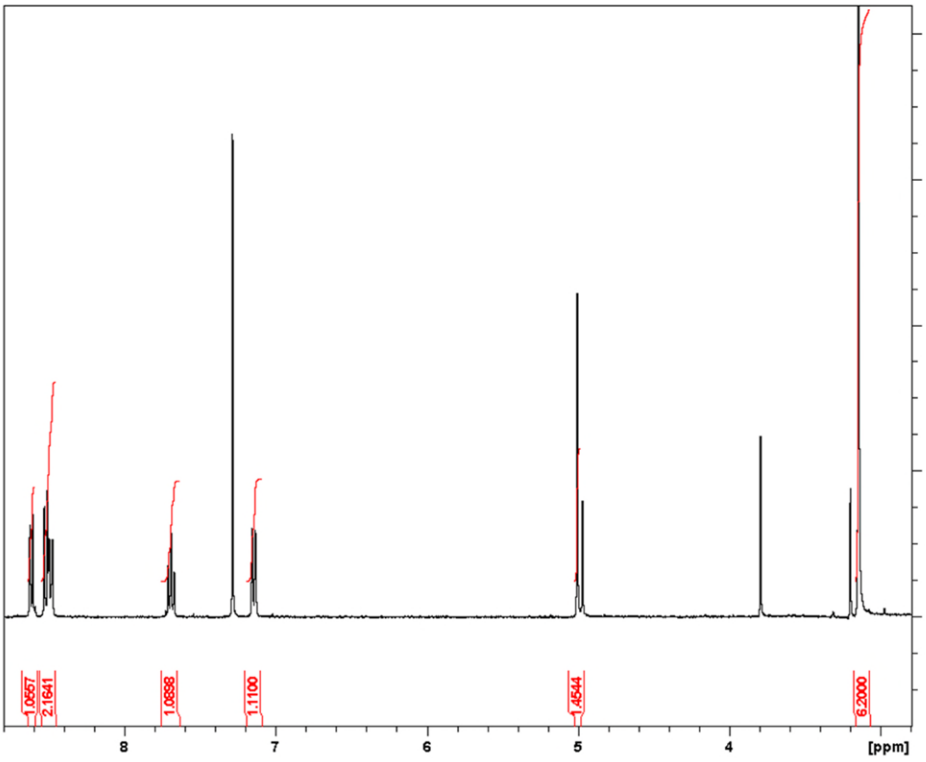

Figure S13. 400 Hz ^{1}H NMR spectrum of ligand **L3** in $CDCl_3$ at 298 K.

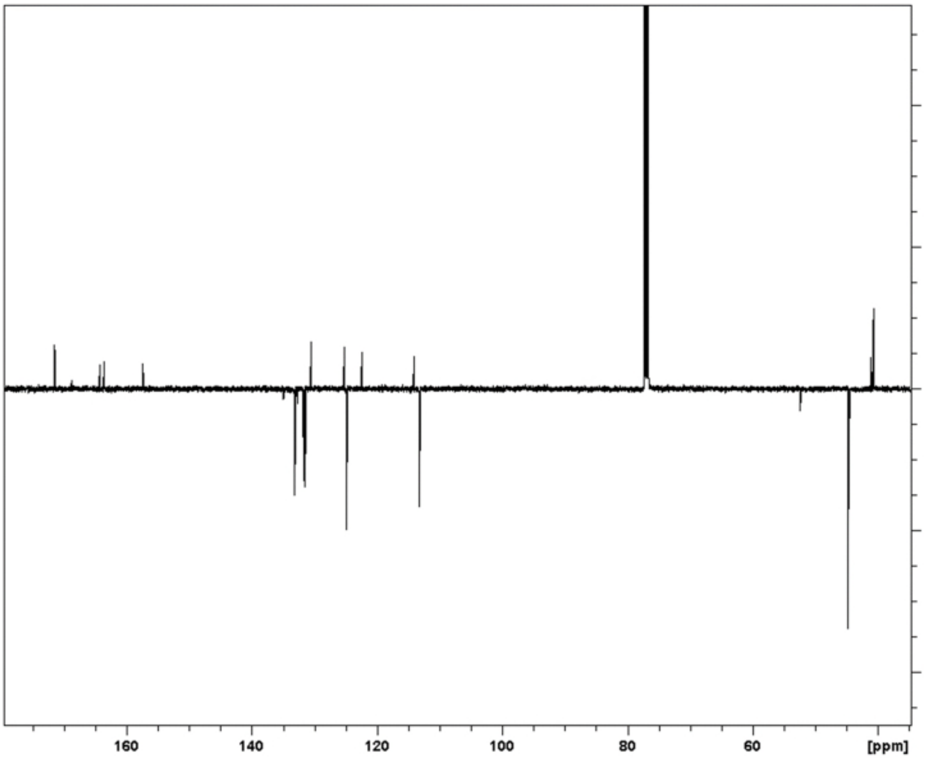

Figure S14. 125 Hz DEPT-135 ^{13}C NMR spectrum of ligand **L3** in $CDCl_3$ at 298 K.

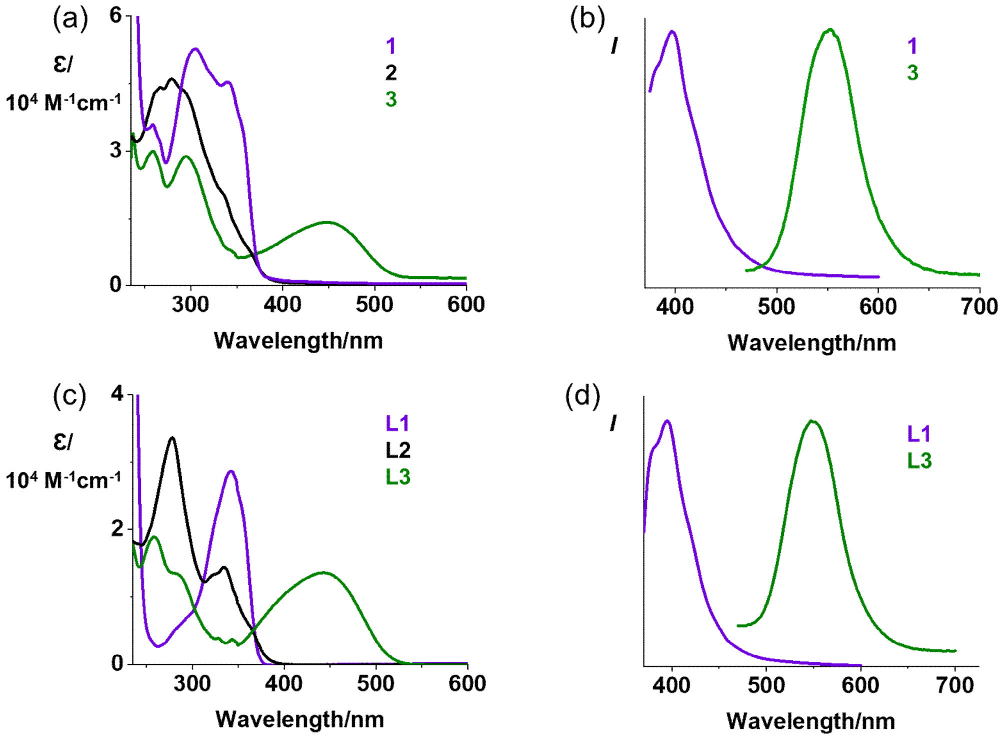

Figure S15. (a) UV-vis absorption spectra and (b) fluorescence spectra of complexes **1–3** and ligands **L1–L3** (c and d) in PBS with 5% DMSO at 298 K. The fluorescence intensity is normalised for comparison. Complex **2** and ligand **L2** are not fluorescent in PBS.

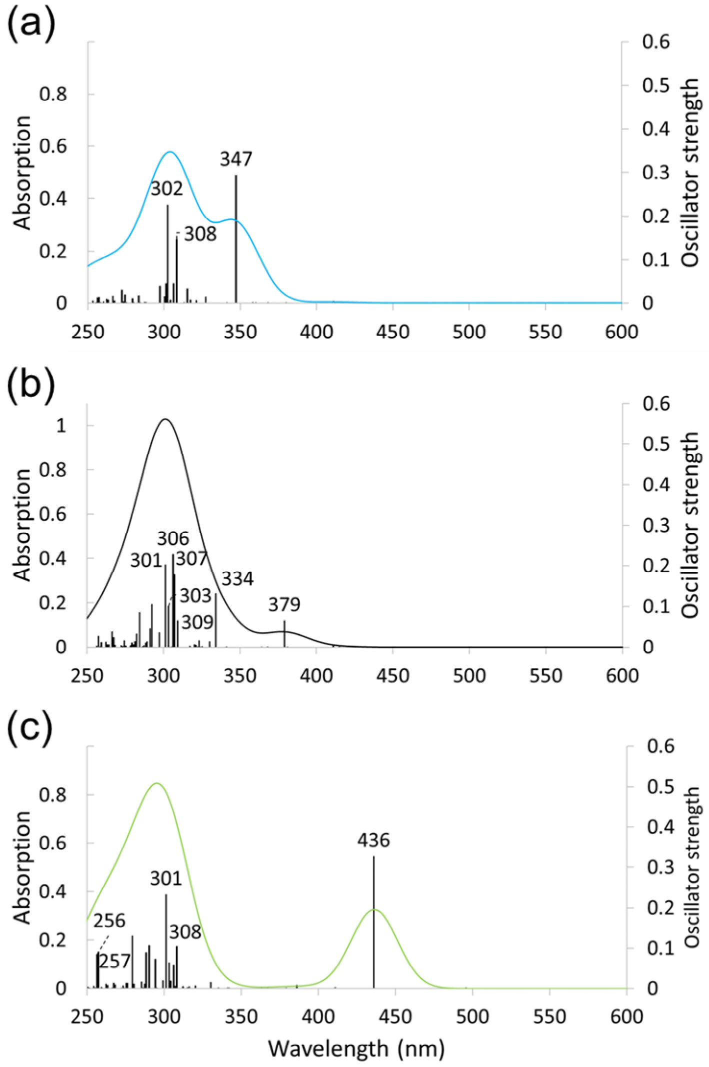

Figure S16. DFT-predicted absorption spectra of complexes **1** (a), **2** (b), and **3** (c), with superposed histograms of the transition oscillator strengths.

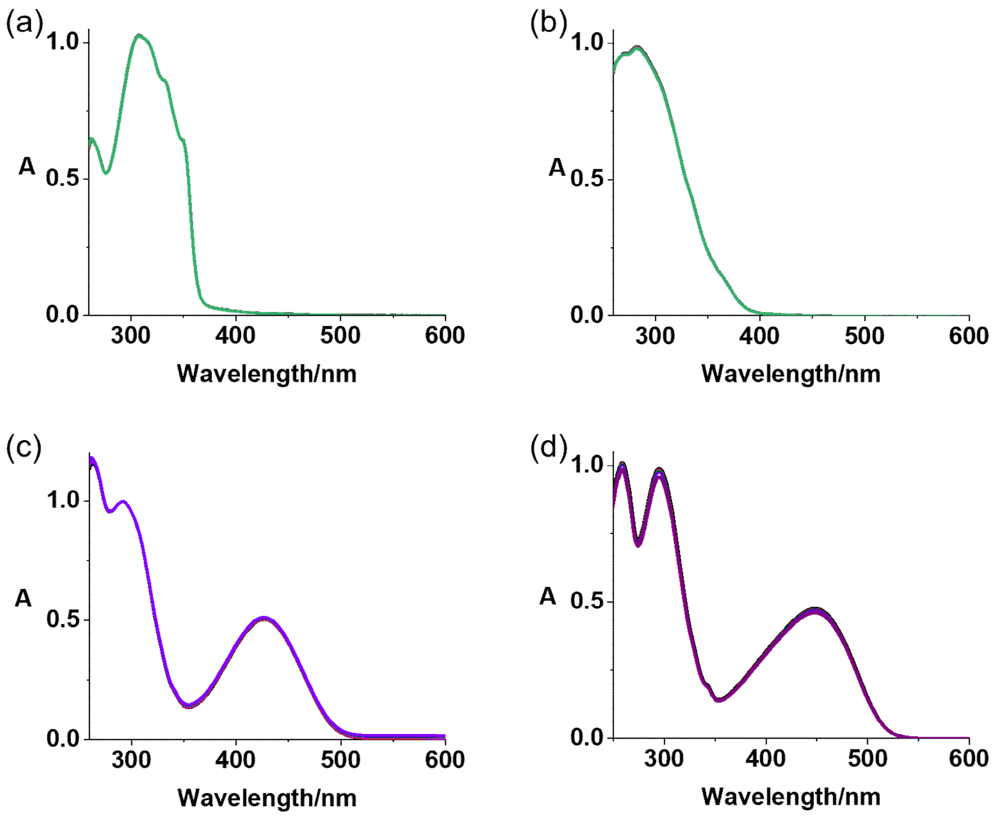

Figure S17. Dark stability of complexes **1** (a), **2** (b), and **3** (c), in DMSO; and (d) **3** in phenol red-free RPMI-1640 with 5% DMSO for 120 min. Spectra were recorded at 0, 20, 40, 90, 120 min.

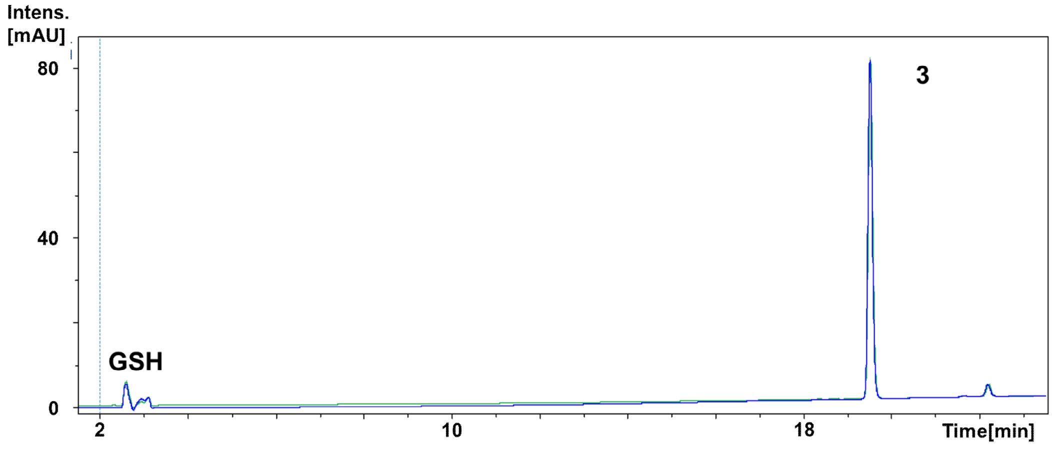

Figure S18. Dark stability of complex **3** (30 μM) in the presence of GSH (2.4 mM) in aqueous solution monitored by HPLC over 120 min.

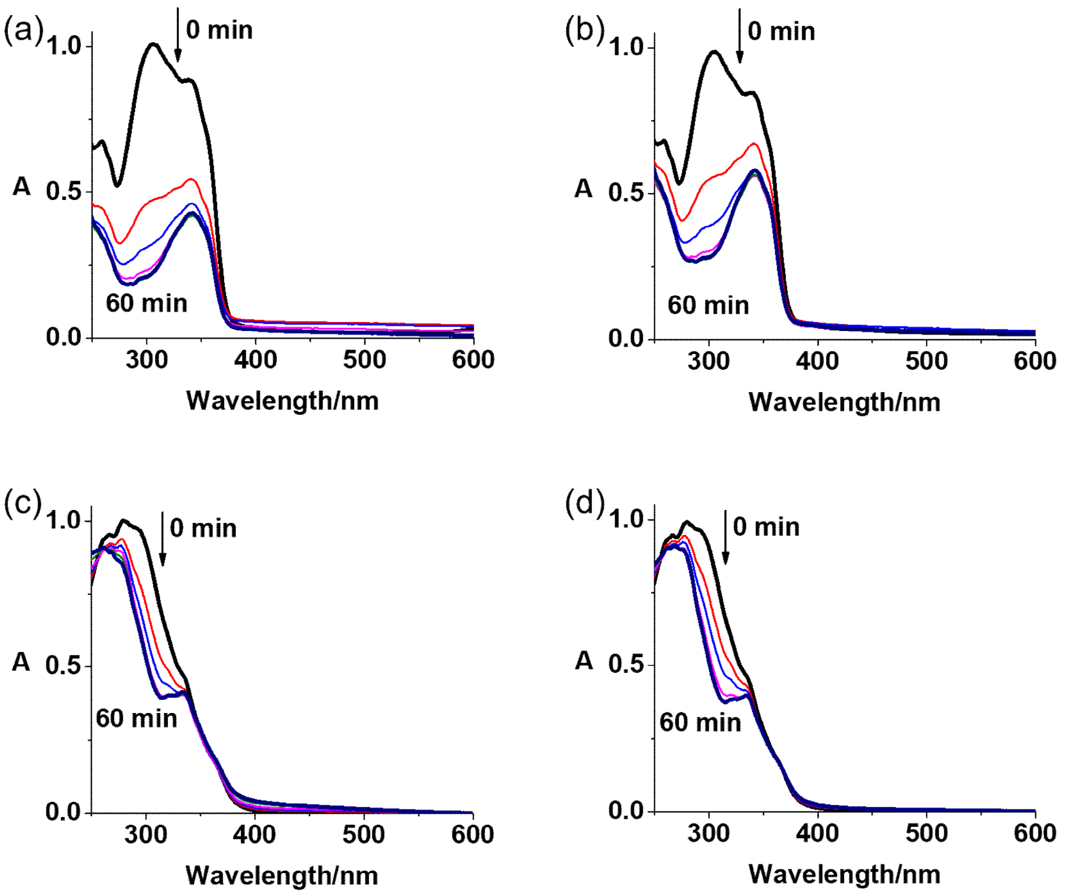

Figure S19. UV-vis absorption spectra showing photochemical decomposition of **1** (a, 420 nm; b, 463 nm) and **2** (c, 420 nm; d, 463 nm) in RPMI-1640 with 5% DMSO, upon irradiation for 60 min. Spectra were recorded at 0, 5, 10, 20, 40 and 60 min.

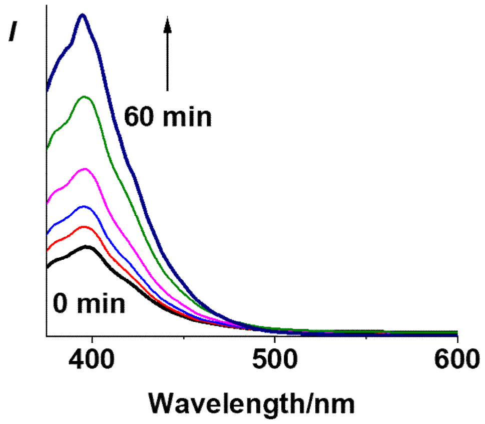

Figure S20. Fluorescence changes during photodecomposition of **1** in PBS with 5% DMSO upon irradiation with blue light (463 nm, spectra were recorded at 0, 5, 10, 20, 40 and 60 min).

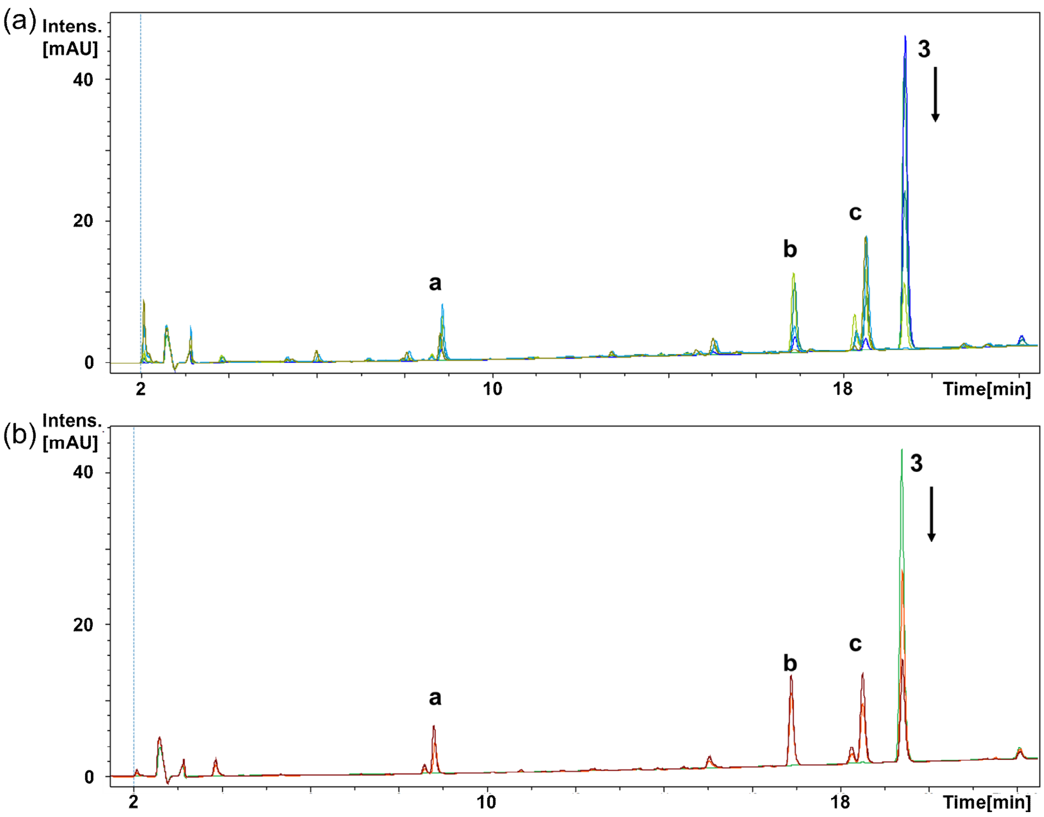

Figure S21. Photochemical decomposition of complex **3** determined by HPLC upon 60 min irradiation with (a) indigo (420 nm, spectra were recorded at 0, 1, 5, 10, 30 and 60 min) and (b) green (517 nm, spectra were recorded at 0, 30 and 60 min) light. a, $\{Pt^{II}(py)_2(CH_3CN)(N_3)\}^+$ (436.00 m/z); b, $\{Pt^{IV}(py)_2(N_3)(OH)(gly\text{-}4\text{-}MMe_2\text{-}Nap)\}^+$ (708.81 m/z); c, gly-4-MMe$_2$-Nap + H^+ (298.48 m/z).

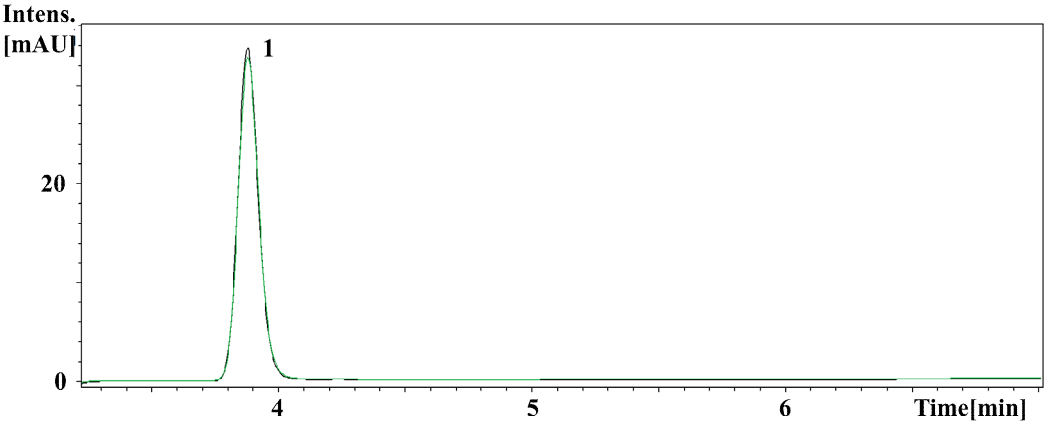

Figure S22. Photochemical decomposition of **FM-190** determined by HPLC upon 60 min irradiation with green light (517 nm, spectra were recorded at 0 and 60 min).

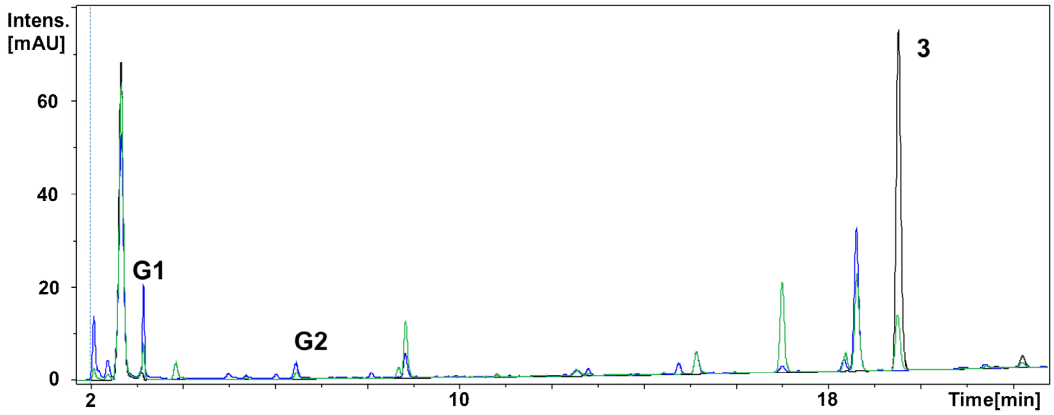

Figure S23. Photoreaction between of **3** and 5’-GMP after 1 h irradiation monitored by HPLC, dark (—), blue light (—), green light (—). G1, $\{Pt^{II}(py)_2(OH)(CH_3CN)\}^+$ (755.73 m/z); G2, $\{Pt^{II}(py)_2(N_3)(GMP)\}^+$ (757.74 m/z).

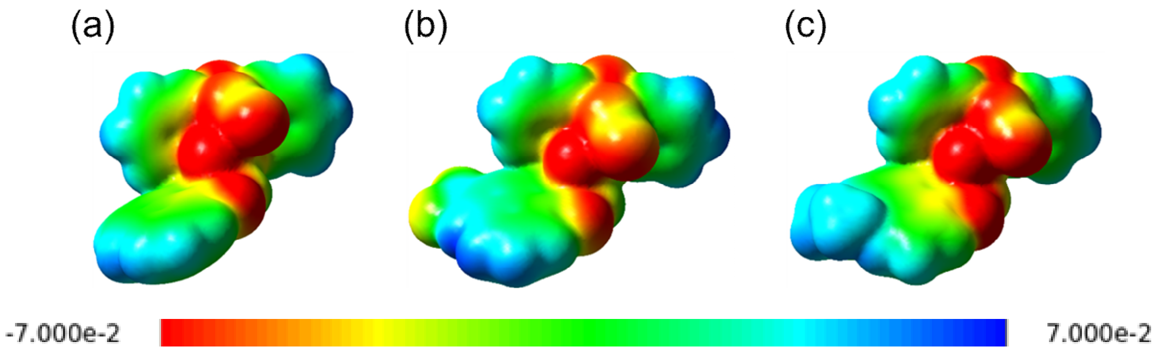

Figure S24. Electrostatic potential mapped on isodensity (n=0.001) surfaces for complex **1** (a), **2** (b), and **3** (c), between -0.07 a.u. (red) and 0.07 a.u. (blue), with intermediate potentials assigned to intermediate colour in the light spectrum.

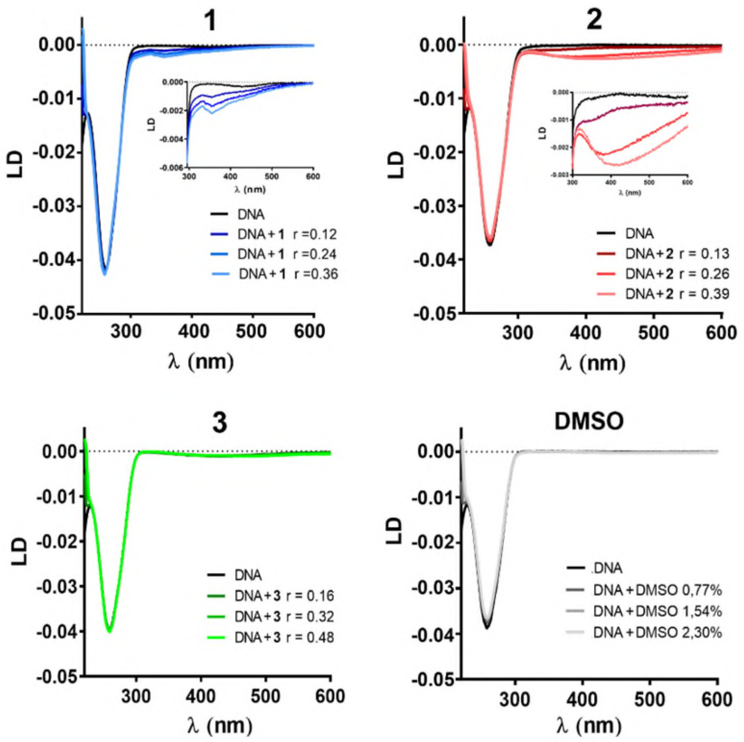

Figure S25. LD spectra of calf thymus DNA in the absence (—) or presence of complexes **1**, **2**, **3**, in 10 mM Tris-Cl, pH 7.4 containing 0.77 – 2.3 v/v DMSO. The DNA concentration was 3×10^{-4} M, and the [Pt]/[DNA] ratios (r) are indicated.

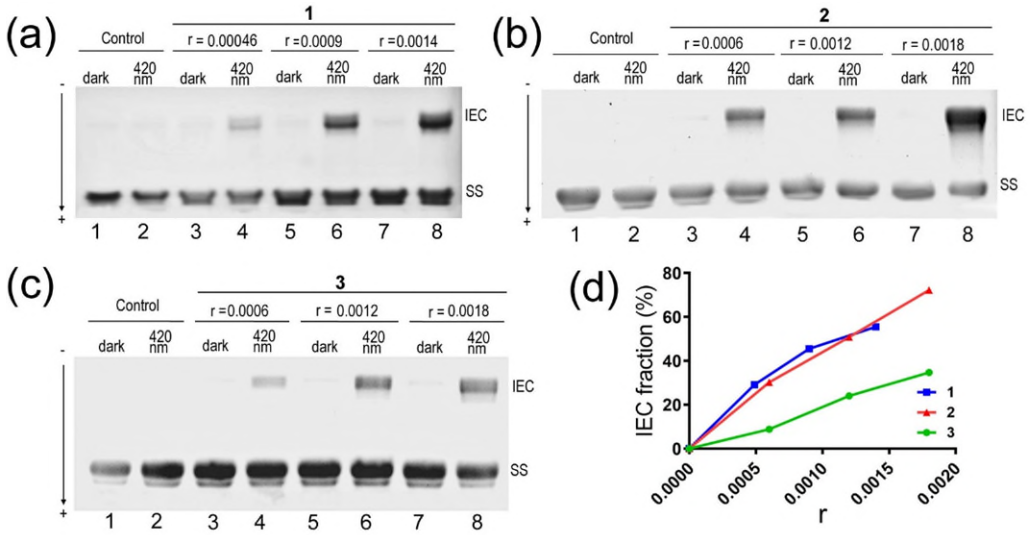

Figure S26. Denaturing agarose gel electrophoresis for determination of DNA interstrand cross-linking by **1** (a), **2** (b), or by **3** (c). Linear plasmid DNA was incubated in the dark or under irradiation at various r as indicated. (d) Quantitative evaluation of the IEC (interstrand cross-links) fraction of the irradiated samples.

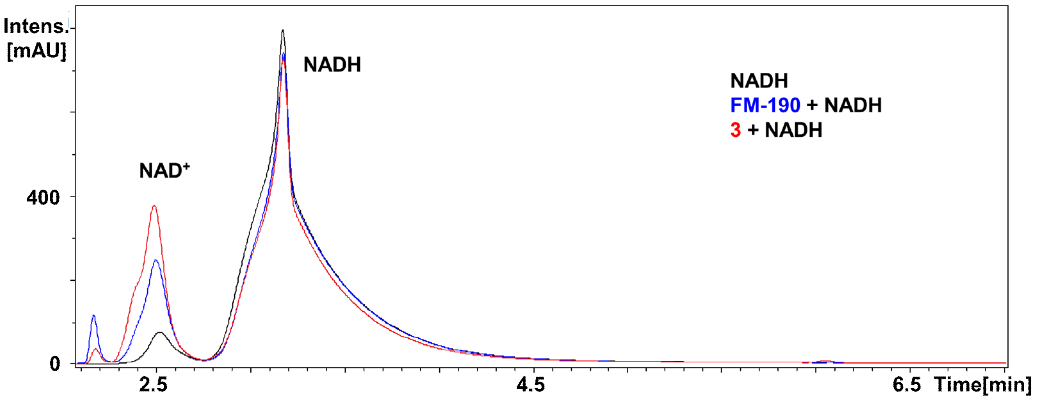

Figure S27. Photooxidation of NADH by complex **3** and **FM-190** after 1 h irradiation with indigo light (420 nm) monitored by HPLC (254 nm), NADH alone was used as reference.

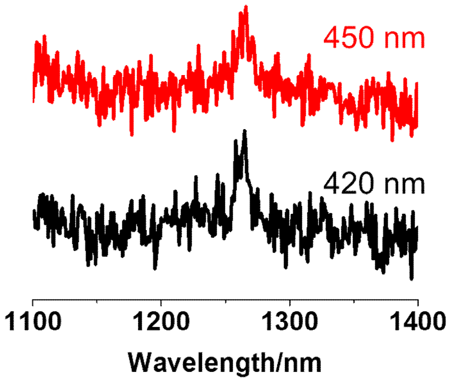

Figure S28. Infrared phosphorescence detection of $^1O_2$ formation when complex **3** (200 µM) in acetonitrile was excited by blue light (420/450 nm).

References

S1. P. Prasad, I. Pant, I. Khan, P. Kondaiah, A. R. Chakravarty, *Eur. J. Inorg. Chem.*, **2014**, *14*, 2420–2431.

S2. N. J. Farrer, J. A. Woods, L. Salassa, Y. Zhao, K. S. Robinson, G. Clarkson, F. S. Mackay, P. J. Sadler, *Angew. Chem. Int. Ed.*, **2010**, *49*, 8905–8908.

S4. S. Liu, Q. Deng, W. Fang, J.-F. Gong, M.-P. Song, M. Xua, T. Tu, *Org. Chem. Front.*, **2014**, *1*, 1261−1265.

S5. I. O. Donkor, Y. S. Abdel-Ghany, P. F. Kador, T. Mizoguchi, A. Bartoszko-Malik, D. D. Miller, *Eur. J. Med. Chem.*, **1999**, *34*, 235−243.

S6. C. S. Swenson, A. Velusamy, H. S. Argueta-Gonzalez, J. M. Heemstra, *J. Am. Chem. Soc.*, **2019**, *141*, 19038–19047.

S7. O. V. Dolomanov, L. J. Bourhis, R. J. Gildea, J. A. K. Howard, H. Puschmann, *J. Appl. Crystallogr.*, **2009**, *42*, 339−341.

S8. G. M. Sheldrick, *Acta Crystallogr., Sect. A: Found. Adv.*, **2015**, *71*, 3−8.

S9. G. M. Sheldrick, *Acta Crystallogr., Sect. C: Struct. Chem.*, **2015**, *71*, 3−8.

S10. Y. Zhao, J. A. Woods, N. J. Farrer, K. S. Robinson, J. Pracharova, J. Kasparkova, O. Novakova, H. Li, L. Salassa, A. M. Pizarro, G. J. Clarkson, L. Song, V. Brabec, P. J. Sadler, *Chem. Eur. J.* **2013**, *19*, 9578 – 9591.

S11. V. Vichai, K. Kirtikara, *Nat. Protoc.*, **2006**, *1*, 1112−1116.

S12. K. Suzuki, A. Kobayashi, S. Kaneko, K. Takehira, T. Yoshihara, H. Ishida, Y. Shiina, S. Oishic, S. Tobita. *Phys. Chem. Chem. Phys.*, **2009**, *11*, 9850– 9860.

S13. H. Shi, Q. Wang, V. Venkatesh, G. Feng, L. S. Young, I. Romero-Canelón, M. Zeng, P. J. Sadler, *Dalton Trans.*, **2019**, *48*, 8560−8564.
